# Source influence on emission pathways and ambient PM_2.5_ pollution over India (2015–2050)

**DOI:** 10.5194/acp-18-8017-2018

**Published:** 2018-06-07

**Authors:** Chandra Venkataraman, Michael Brauer, Kushal Tibrewal, Pankaj Sadavarte, Qiao Ma, Aaron Cohen, Sreelekha Chaliyakunnel, Joseph Frostad, Zbigniew Klimont, Randall V. Martin, Dylan B. Millet, Sajeev Philip, Katherine Walker, Shuxiao Wang

**Affiliations:** 1 Department of Chemical Engineering, Indian Institute of Technology Bombay, Powai, Mumbai, India; 2 Interdisciplinary program in Climate Studies, Indian Institute of Technology Bombay, Powai, Mumbai, India; 3 School of Population and Public Health, The University of British Columbia, Vancouver, British Columbia V6T1Z3, Canada; 4 Institute for Advanced Sustainability Studies (IASS), Berliner Str. 130, 14467 Potsdam, Germany; 5 State Key Joint Laboratory of Environment Simulation and Pollution Control, School of Environment, Tsinghua University, Beijing 100084, China; 6 Health Effects Institute, Boston, MA 02110, USA; 7 Department of Soil, Water, and Climate, University of Minnesota, Minneapolis–Saint Paul, MN 55108, USA; 8 Institute for Health Metrics and Evaluation, University of Washington, Seattle, WA 98195, USA; 9 International Institute for Applied Systems Analysis, Laxenburg, Austria; 10 Department of Physics and Atmospheric Science, Dalhousie University, Halifax, Nova Scotia B3H 4R2, Canada; 11 NASA Ames Research Center, Moffett Field, California, USA; 12 State Environmental Protection Key Laboratory of Sources and Control of Air Pollution Complex, Beijing 100084, China

## Abstract

India is currently experiencing degraded air quality, and future economic development will lead to challenges for air quality management. Scenarios of sectoral emissions of fine particulate matter and its precursors were developed and evaluated for 2015–2050, under specific pathways of diffusion of cleaner and more energy-efficient technologies. The impacts of individual source sectors on PM_2.5_ concentrations were assessed through systematic simulations of spatially and temporally resolved particulate matter concentrations, using the GEOS-Chem model, followed by population-weighted aggregation to national and state levels. We find that PM_2.5_ pollution is a pan-India problem, with a regional character, and is not limited to urban areas or megacities. Under present-day emissions, levels in most states exceeded the national PM_2.5_ annual standard (40 μg m^−3^). Sources related to human activities were responsible for the largest proportion of the present-day population exposure to PM_2.5_ in India. About 60 % of India’s mean population-weighted PM_2.5_ concentrations come from anthropogenic source sectors, while the remainder are from “other” sources, windblown dust and extra-regional sources. Leading contributors are residential biomass combustion, power plant and industrial coal combustion and anthropogenic dust (including coal fly ash, fugitive road dust and waste burning). Transportation, brick production and distributed diesel were other contributors to PM_2.5_. Future evolution of emissions under regulations set at current levels and promulgated levels caused further deterioration of air quality in 2030 and 2050. Under an ambitious prospective policy scenario, promoting very large shifts away from traditional biomass technologies and coal-based electricity generation, significant reductions in PM_2.5_ levels are achievable in 2030 and 2050. Effective mitigation of future air pollution in India requires adoption of aggressive prospective regulation, currently not formulated, for a three-pronged switch away from (i) biomass-fuelled traditional technologies, (ii) industrial coal-burning and (iii) open burning of agricultural residue. Future air pollution is dominated by industrial process emissions, reflecting larger expansion in industrial, rather than residential energy demand. However, even under the most active reductions envisioned, the 2050 mean exposure, excluding any impact from windblown mineral dust, is estimated to be nearly 3 times higher than the WHO Air Quality Guideline.

## 1 Introduction

India hosts the world’s second largest population (UNDP, 2017), but accounts for only 6 % of the world’s total primary energy use (IEA, 2015). However, India is an emerging economy with significant growth in a multitude of energy-use activities in industry and transport sectors, as well as in residential, agricultural and informal industry sectors (Sadavarte and Venkataraman, 2014; Pandey et al., 2014). With expansion in power generation (CEA, 2016) and industrial production (Planning Commission, Government of India, 2013), emissions from these sectors were estimated to have increased about 2-fold between 1995 and 2015 (Sadavarte and Venkataraman, 2014). There is a steady demand for motorized vehicles for both personal and public transport, with an increase in ownership of motorized two-wheeler motorcycles and scooters and four-wheeler cars (MoRTH, 2012), in both rural and urban areas. Traditional technologies, and the use of solid biomass fuels, are widespread in the residential sector (cooking with biomass fuel cook stoves and lighting with kerosene wick lamps), the agricultural sector (open burning of agricultural residue for field clearing) and the informal industry sector (brick production, processing of food and agricultural products). Ambient PM_2.5_ (particulate matter in a size fraction with aerodynamic diameter smaller than 2.5 μm) concentrations are influenced by emissions of both primary or directly emitted PM_2.5_, and its precursor gases, including SO_2_, NH_3_, NO_*x*_ and NMVOCs (non-methane volatile organic compounds), whose atmospheric reactions yield secondary particulate sulfate, nitrate and organic carbon, while reactions of NO_*x*_ and NMVOCs also increase ozone levels. Ozone precursor gases and particulate black carbon and organic carbon (BC and OC) are identified in the list of short-lived climate pollutants or SLCPs (CCAC, 2014).

Air quality is a public health issue of concern in India. According to the World Health Organization (WHO), 37 cities from India feature in a global list of 100 world cities with the highest PM_10_ (PM with aerodynamic diameter < 10 μm) pollution, with cities like Delhi, Raipur, Gwalior and Lucknow listed among the world’s top 10 polluted cities (WHO, 2014; further details in Fig. S6 in the Supplement). Recent studies (Ghude et al., 2016; Chakraborty et al., 2015) have built upon products of the Task Force on Hemispheric Transport of Air Pollutants (TF-HTAP), using HTAP emission inventories (for 2010) in a regional chemistry model to address air quality in India. Widespread PM_2.5_ and O_3_ pollution was found under present-day emission levels, which considerably impact human mortalities and life expectancy. To extend the understanding of ambient air pollution to multiple (regional and national) scales, for multiple pollutants, methods which combine chemical transport modelling with data from satellite retrievals combined with available monitoring data have been developed (van Donkelaar et al., 2010; Brauer et al., 2012, 2016; Dey et al., 2012; Shaddick et al., 2018) and can be used to evaluate current levels and trends. The latest Global Burden of Disease (GBD) 2015 estimates indicate that the population-weighted mean PM_2.5_ concentration for India as a whole was 74.3 μg m^−3^ in 2015, up from about 60 μg m^−3^ in 1990 (Cohen et al., 2017). At current levels, 99.9 % of the Indian population is estimated to live in areas where the World Health Organization (WHO) Air Quality Guideline of 10 μg m^−3^ was exceeded. Nearly 90 % of people lived in areas exceeding the WHO Interim Target 1 of 35 μg m^−3^.

Strategies for mitigation of air pollution require understanding of pollutant emissions, differentiated by emitting sectors and by sub-national regions, representing both present-day conditions and future evolution under different pathways of growth and technology change. Future projections of emissions, for climate relevant species, are available in the representative concentration pathway (RCP) scenarios (Fujino et al., 2006; Clarke et al., 2007; van Vuuren et al., 2007; Riahi et al., 2007; Hijioka et al., 2008), more recently for the Shared Socioeconomic Pathway (SSP) scenarios (Riahi et al., 2017; Rao et al., 2017), while primary PM_2.5_ is included in inventories like ECLIPSE (Klimont et al., 2017, 2018). Inventories developed for HTAP_v2 (Janssens-Maenhout et al., 2015) address emissions of a suite of pollutants for 2008 and 2010. These scenarios and emission datasets are developed through globally consistent methodologies, leaving room for refinement through more detailed regional studies. Thus, in this work we develop and evaluate sectoral emission scenarios of fine particulate matter and its precursors and constituents from India, during 2015–2050, under specific pathways of diffusion of cleaner and more energy-efficient technologies. The work is broadly related to HTAP scientific questions including understanding of (i) sensitivity of regional PM_2.5_ pollution levels to magnitudes of emissions from source sectors and (ii) changes in PM_2.5_ levels as a result of expected, as well as ambitious, air pollution and climate change abatement efforts. The impacts of individual source sectors on PM_2.5_ concentrations is assessed through simulation of spatially and temporally resolved particulate matter concentrations, using the GEOS-Chem chemical transport model, followed by aggregation to population-weighted concentrations (estimated as the sum of product of concentration and population for each grid divided by the total population) at both national and state levels.

Section 2 discusses the development of the emission inventory, disaggregated by sector, for the year 2015 and future projections to 2050; Sect. 3 describes the GEOS-Chem model, the simulation parameters and evaluation; Sect. 4 discusses simulated PM_2.5_ concentration by sector, at national and state levels under present-day and future emission scenarios; and the last section discusses findings and conclusions.

## 2 Present-day and future emissions

### 2.1 Present-day emissions (2015)

An emission inventory was developed for India for the year 2015, based on an engineering model approach using technology-linked energy-emissions modelling adapted from previous work (Pandey and Venkataraman, 2014; Pandey et al., 2014; Sadavarte and Venkataraman, 2014), to estimate multi-pollutant emissions including those of SO_2_, NO_*x*_, PM_2.5_, black carbon (BC), organic carbon (OC) and non-methane volatile organic compounds (NMVOCs). An engineering model approach goes beyond fuel divisions and uses technology parameters for process and emissions control technologies, including technology type, efficiency or specific fuel consumption and technology-linked emission factors (g of pollutant/kg of fuel) to estimate emissions.

The inventory disaggregates emissions from technologies and activities in all major sectors. Plant-level data (installed capacity, plant load factor and annual production) are used for 830 individual large point sources, in heavy industry and power generation sectors, while light industry activity statistics (energy consumption, industrial products, solvent use, etc.) are from a sub-state (or district) level (CEA, 2010; CMA, 2007a, b, 2012; MoC, 2007; FAI, 2010; CMIE, 2010; MoPNG, 2012; MoWR, 2007). Technology-linked emission factors and current levels of deployment of air pollution control technologies are used. Vehicular emissions include consideration of vehicle technologies, vehicle age distributions and super-emitters among on-road vehicles (Pandey and Venkataraman, 2014). Residential sector activities comprise cooking and water heating, largely with traditional biomass stoves, lighting, using kerosene lamps and warming of homes and humans, using biomass fuels. Seasonality in emissions from the residential sector is considered for water heating and home warming. The “informal industries” sector includes brick production (in traditional kiln technologies like Bull’s trench kilns and clamp kilns, using both coal and biomass fuels) and food and agricultural product processing operations (like drying and cooking operations related to sugarcane juice, milk, food grain, jute, silk, tea and coffee). In addition, monthly mean data on agricultural residue burning in fields, a spatio-temporally discontinuous source of significant emissions, were calculated using a bottom-up methodology (Pandey et al., 2014). Spatial proxies used to estimate gridded emissions over India are described in Table S1 in the Supplement.

**Figure 1.**
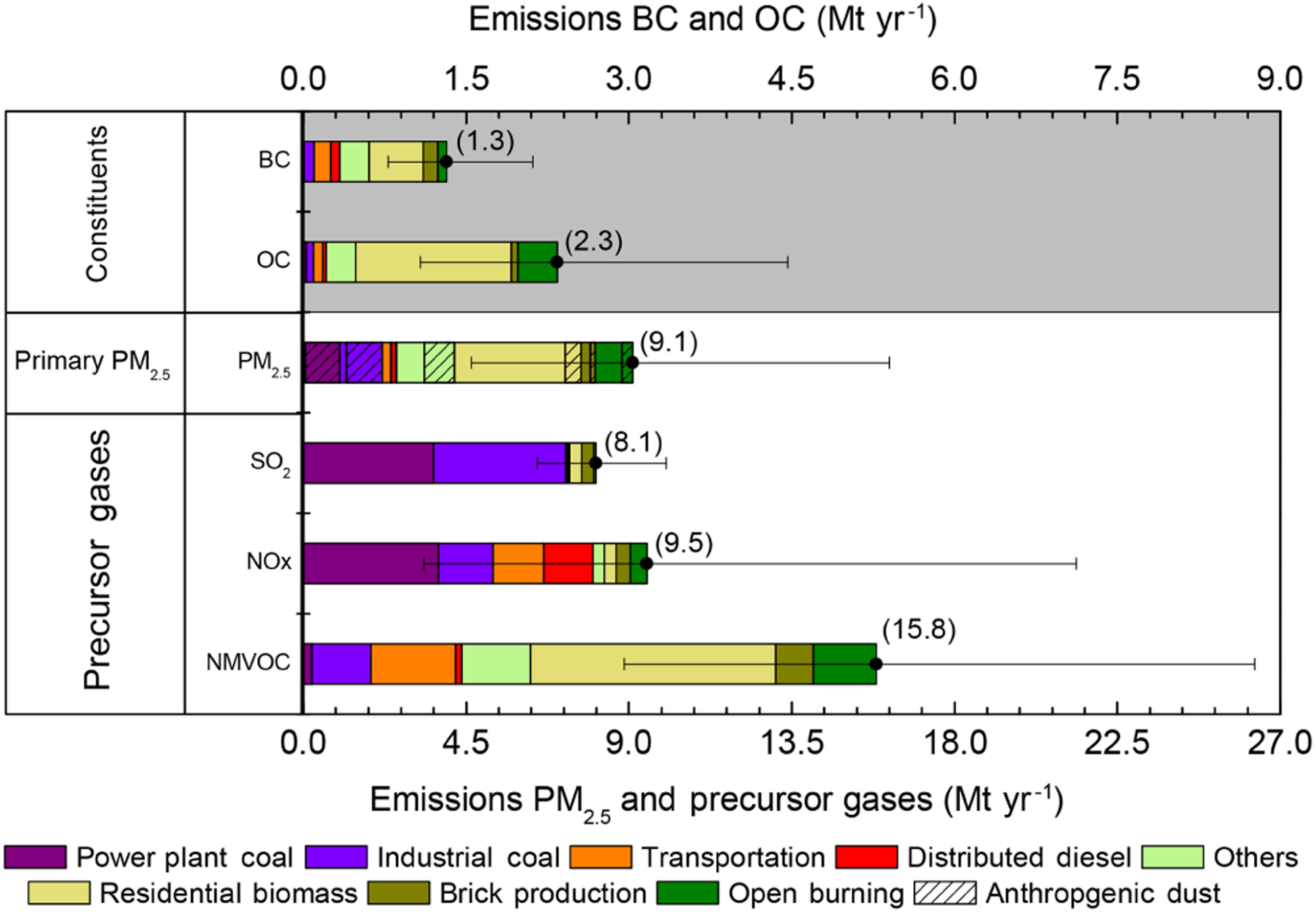
National emissions of particulate matter and precursor gases for 2015 (Mt yr^−1^). Emissions of NO_*x*_ are in Mt yr^−1^ of NO and emissions of SO_2_ are in Mt yr^−1^ of SO_2_.

India emissions for 2015 of PM_2.5_, BC, OC, SO_2_, NO_*x*_, and NMVOCs by sector (Fig. 1) arose from three main sources: (i) residential biomass fuel use (for cooking and heating), (ii) coal burning in power generation and heavy industry and (iii) open burning of agricultural residue for field clearing. Table 1 provides a description of sectors and constituent source categories. Emissions linked to incomplete fuel combustion, including PM_2.5_ (9.1 Mt yr^−1^, or million tonnes per year), BC (1.3 Mt yr^−1^), OC (2.3 Mt yr^−1^) and NMVOCs (33.4 Mt yr^−1^), arose primarily from traditional biomass technologies in the residential sector (for cooking and heating) and the informal industry sector (for brick production and for food and agricultural produce processes), as well as from agricultural residue burning. Emissions of SO_2_ (8.1 Mt yr^−1^) and NO_*x*_ (9.5 Mt yr^−1^) arose largely from coal boilers in industry and power sectors and from vehicles in the transport sector. Emissions of CO are included in the inventory (Pandey et al., 2014; Sadavarte and Venkataraman, 2014); however, CO was not input in the GEOS-Chem simulations, since it is not central to atmospheric the chemistry of secondary PM_2.5_ formation on annual timescales.

Detailed tabulations of 2015 emissions of each pollutant at the state level are provided in Table S2 in the Supplement. Uncertainties in the activity rates, calculated analytically using methods described more fully in previous publications (Pandey and Venkataraman, 2014; Pandey et al., 2014; Sadavarte and Venkataraman, 2014), are shown in Table S3 in the Supplement.

### 2.2 Future emission pathways (2015–2050)

#### 2.2.1 Description of future emission scenarios

We develop and evaluate three future scenarios which extend from 2015 to 2050, which are likely to bound the possible amplitude of future emissions, based on the expected future evolution of sectoral demand, following typical methods in previous studies (Cofala et al., 2007; Ohara et al., 2007). These include a reference (REF) scenario and two scenarios (S2 and S3) representing different levels of deployment of high-efficiency, low-emissions technologies (Table 2). The scenarios capture varying levels of emission control, with no change in current (2015) regulations, corresponding to very slow uptake of new technology (REF), adoption of promulgated regulations, corresponding to effective achievement of targets (S2) and adoption of ambitious prospective regulations, corresponding to those well beyond promulgated regulations (S3). In both S2 and S3, despite expanding sectoral demand, there is reduced energy consumption from adoption of clean energy technologies, at different levels.

The methodology for emission projection includes estimation of future evolution in (i) sectoral demand, (ii) technology mix, (iii) energy consumption and (iv) technology-linked emission factors (Fig. S1 in the Supplement). Activity levels in future years by source category (e.g. GWh installed capacity in power, vehicle-kilometres travelled in transport, industrial production, e.g. in tons, population of users in residential areas) were apportioned to various technology divisions, using an assumed evolving technology mix, for three different scenarios. Activity at the technology division level was used to derive corresponding future energy (and fuel) consumption and related emissions using technology-based emission factors.

With 2015 as the base year, growth rates in sectoral demand were identified for thermal power plants, industries, residential, brick kilns and informal industries, on-road transportation and agricultural sectors for 2015–2030 and 2030–2050 (Table S4 in the Supplement). Sectoral growth levels, estimated as ratios of 2050 to 2015 demand, were 5.1, 3.8, 3.2, 1.3 and 1.4 respectively, for the building sector, electricity generation, heavy industries, residential sector and agricultural residue burning, the largest growth being in the building and electricity generation sectors (Fig. S2 in the Supplement).

Table 2 shows regulation levels for different sectors under the three scenarios, through to 2050. The REF and S2 scenarios capture both energy efficiency and emissions control, continuing under current regulation, or broadly under promulgated future policies. The S2 scenario assumes shifts to non-fossil generation which would occur under India’s Nationally Determined Contribution (India’s NDC, 2015) in the power sector, consistent with a shift to 40 % renewables including solar, wind and hydropower by 2030 (India’s NDC, 2015). The NDC goals of India are suggested to be realistic (CAT, 2017; Ross and Gerholdt, 2017), with achievement of non-fossil share of power generation projected to lie between 38 and 48 % by 2030, as well as adoption of tighter emission standards for desulfurization and de-NO_*x*_ technologies in thermal plants (MoEFCC, 2015), at a rate consistent with expected barriers (CSE, 2016). Further, changes assumed in the transport sector reflect promulgated growth in public vehicle share (NTDPC, 2013; Guttikunda and Mohan, 2014; NITI Aayog, 2015) and promulgated regulation (Auto Fuel Policy Vision 2025, 2014; MoRTH, 2016), along with realistic assumptions of implementation lags in adoption of BS VI standards (ICRA, 2016). Other assumptions include modest increases in industrial energy efficiency under the Perform Achieve and Trade (PAT) scheme (Level 2, IESS, Niti Aayog, 2015), modest increases in non-fired-brick walling materials (UNDP, 2009; Sameer Maithel, personal communication, 2016), a slow shift to more efficient residential energy technologies and fuels (Level 2, IESS, Niti Aayog, 2015) and a minor reduction in agricultural residue burning.

However, in the S3 scenario, adoption of ambitious regulations, well beyond those currently promulgated, is assumed. This includes very significant shifts to non-fossil-power generation (Anandarajah and Gambhir, 2014; Shukla and Chaturvedi, 2012; Level 4, IESS, Niti Aayog, 2015), a near-complete shift to high-efficiency industrial technologies (MoP, 2012, Level 4, IESS, Niti Aayog, 2015), a large public vehicle share (NITI Aayog, 2015), energy efficiency improvements in engine technology (MoP, 2015), a large share of electric and compressed natural gas (CNG) vehicles (NITI Aayog, 2015), a complete switch to LPG/PNG or biogas or high-efficiency gasifier stoves for residential cooking and heating (Level 4, IESS, Niti Aayog, 2015) and to solar and electric lighting (National Solar Mission, 2010) by 2030 and a significant (by 2030) and complete (by 2050) phase-out of agricultural residue burning, through a switch to mulching practices (Gupta, 2014). Further details of the shift in technologies can be found in Table S5 and related discussion in the Supplement (see Sect. S2.3).

As alluded to earlier, there is a reduction in total energy consumption in future years, despite increase in activity, in scenarios S2 and S3, which assumes the large deployment of high-efficiency energy technologies. The projected energy demand under the three scenarios (Fig. S3, Sect. S2.4) is in general agreement with published work (Anandarajah and Gambhir, 2014; Chaturvedi and Shukla, 2014; Parikh, 2012; Shukla et al., 2009), 95 to 110 EJ for reference scenarios (Parikh, 2012; Shukla and Chaturvedi, 2012) and 45–55 EJ for low carbon pathways (Anandarajah and Gambhir, 2014; Chaturvedi and Shukla, 2014) in 2050. Projections of CO_2_ emissions to 2050, of 7200 Mt yr^−1^ in REF and 2000 Mt yr^−1^ in S3, are broadly consistent with published 2050 values of 7200–7800 Mt yr^−1^ CO_2_ for reference cases and 2500–3400 Mt yr^−1^ CO_2_ under different low carbon scenarios (Anandarajah and Gambhir, 2014; Shukla et al., 2009).

Technology-based emission factors, for over 75 technology/activity divisions, are described in previous publications (Pandey et al., 2014; Sadavarte and Venkataraman, 2014). In addition to fuel combustion, emissions are estimated from industrial “process” activities predominant in industries such as those producing cement and non-ferrous metals, and refineries producing iron and steel (Table S8, Sect. S2.5). In fired-brick production, recently measured emission factors for this sector of PM_2.5_, BC and OC (Weyant et al., 2014) are used (Table S8 in the Supplement), while for gases, in the absence of measurements from brick kilns, those of coal stokers are used. In the transport sector, emission factors for seven categories of vehicles, across two vintage classes, were applied to a modelled on-road vehicle age distribution (Pandey and Venkataraman, 2014). For future emissions, recommendations to adopt stringent emission standards and shift to alternate fuels are taken from the Auto Fuel Policy 2025 (Auto Fuel Vision and Policy 2025, 2014) and measures are accounted for to leapfrog directly to BS-VI for all on-road vehicle categories (MoRTH, 2016). To be consistent with our scenario descriptions, the REF scenario still takes into account the BS-V standards for 2030 and 2050, while the effect of dynamic policy reforms is reflected in the mix of technology in S2 and S3 scenarios by assuming different levels of BS-VI. The share of BS-VI is kept at modest levels, owing to a delay in availability of BS-VI compliant fuels and difficulties in making the technologies adaptive to Indian road conditions as well as cost-effective (ICRA, 2016); however, this would not affect emission factors significantly (Table S8 in the Supplement).

#### 2.2.2 Estimated emission evolution (2015–2050)

The net effect of scenario-based assumptions is that under the REF scenario, emissions are projected to increase steadily over time. Under the S2 scenario, they are also projected to increase but at a slower rate. Only under the most ambitious scenario, S3, are appreciable reductions in emissions of the various air pollutants expected.

**Figure 2.**
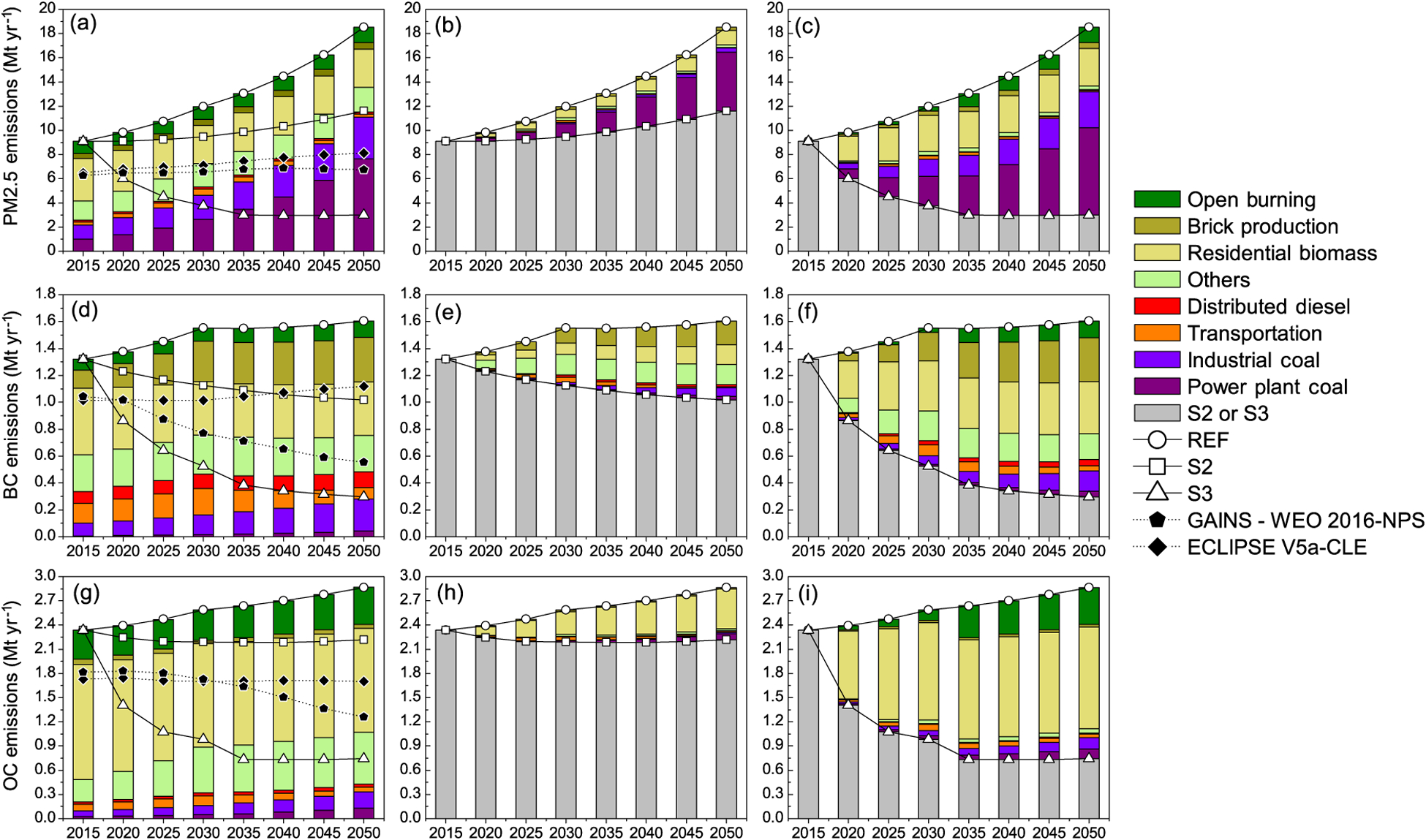
Sectoral emission of fine **(a)** particulate matter, **(d)** black carbon and **(g)** organic carbon under the three scenarios, for 2015–2030 (column 1). Differences of higher efficiency/emission control scenarios from reference (S2 & S3) are shown in column 2 **(b, e, h)** and column 3 **(c, f, i)**. Emissions from ECLIPSE V5a-CLE and GAINS-WEO2016-NPS are shown for comparison.

**Figure 3.**
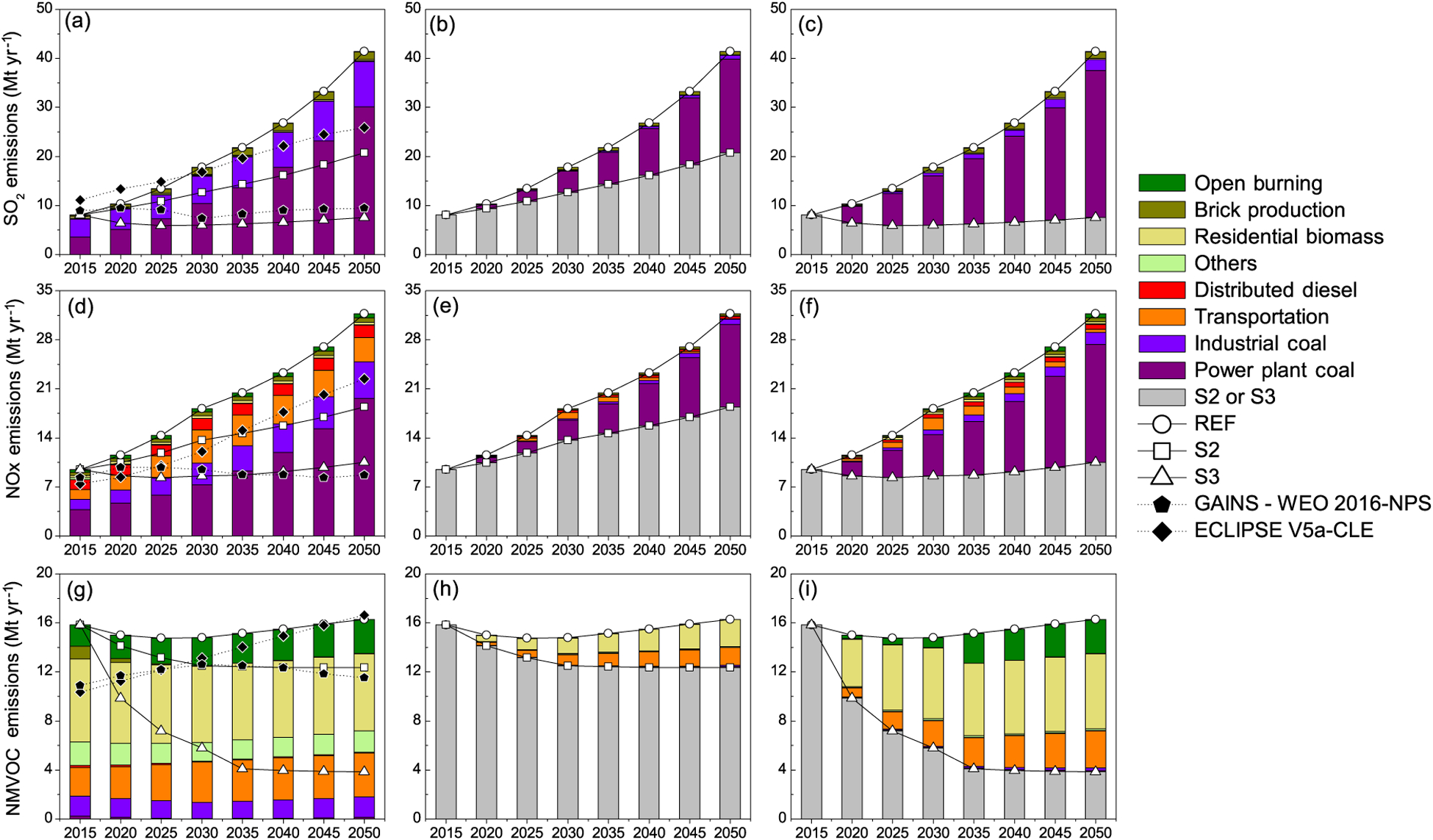
Sectoral emission of fine **(a)** SO_2_, **(d)** NO_*x*_ and **(g)** NMVOCs under the three scenarios, for 2015–2030 (column 1). Differences of higher efficiency/emission control scenarios from reference (REF & S2) are shown in column 2 **(b, e, h)** and column 3 **(c, f, i)**. Emissions from ECLIPSE V5a-CLE and GAINS-WEO2016-NPS are shown for comparison.

Emissions of PM_2.5_ evolve from present-day levels of 9.1 Mt yr^−1^ to 2050 levels of 18.5, 11.5 and 3.0 Mt yr^−1^, respectively, in the three scenarios (Fig. 2a, b, c). These arise from three main sources: (i) traditional biomass technologies in residential, brick production and informal industry, (ii) coal burning in power generation and heavy industry and (iii) open burning of agricultural residue for field clearing. In Figs. 1–3, emissions shown are only from agricultural burning, while those from forest and wildfires, taken from global products, described later, are input to the simulations. In all future scenarios, there is faster growth of industry and electricity generation than of residential energy demand; the former contributes nearly 60–70 % of future emissions. Thus, controlling emissions of PM_2.5_ should come from these sectors. As is quite evident (Fig. 2b and c), assuming large shifts to non-coal power generations in scenarios S2 (40–60 %) and S3 (75–80 %) contributes most to reductions in future emissions of PM_2.5_. Further reductions in emissions are obtained through shifts to cleaner technology and fuels in the residential sector, such as the use of gasifiers and LPG for cooking, electricity and solar devices for lighting and heating and a complete phase-out of open burning of agricultural waste. Black carbon and co-emitted organic carbon have very similar sources, with the largest emissions arising from traditional biomass technologies in the residential and informal industry sectors and from agricultural field burning. Future reductions in BC (Fig. 2d, e, f) and OC (Fig. 2g, h, i) emissions result from a number of policies addressing residential and informal industry sectors as well as agricultural practices. These include actions that enable a shift to cleaner residential energy solutions and a shift away from fired-brick walling materials toward a greater use of clean brick production technologies, as well as a shift away from agricultural field burning through the introduction of mulching practices (assumed in S3). Future increases in transport demand could lead to increased BC emissions from diesel-powered transport, thus providing an important decision lever in favour of the introduction of CNG or non-fossil-electricity-powered public transport (in S3). While diesel particle filters provide a technology for diesel PM and BC control, challenges remain, including the supply of low-sulfur fuel and compliance with NO_*x*_ emission standards.

Emissions of SO_2_ increase in 2050 (Fig. 3d, e, f) to 41.4–20.7 Mt yr^−1^ under REF and S2, but stabilize at 7.5 Mt yr^−1^ under S3. Under both REF and S2 scenarios (Fig. 3a, b, c), emission growth of SO_2_ is driven by growth in electricity demand and industrial production, while reduction is driven by a shift to non-carbon power generation (nuclear, hydro, solar and wind) and modest adoption of flue gas desulfurization technology. In December 2015, the Indian Ministry of Environment and Forests issued new norms for thermal plants with emission standards for SO_2_ and NO_*x*_ (MoEFCC, 2015). Our assumption here of negligible flue gas desulfurization technology results from reported barriers to the adoption of desulfurization and de-NO_*x*_ technologies (CSE, 2016). Little progress was found (CSE, 2016) in the implementation of new standards, from a lack of technology installation/operation information, space for retrofitting and clarity on cost recovery. Transport-related SO_2_ emissions are negligible in all scenarios. Emissions of NO_*x*_ increase in 2050 (Fig. 3d, e, f) to 31.7–18.4 Mt yr^−1^ under REF and S2, but stabilize at 10.5 Mt yr^−1^ under S3. The emissions shares are dominated by thermal power and the transport sector, and grow with sectoral growth under the first two scenarios. Under future scenarios, the demand in passenger-kilometres increases to twice that of ton-kilometres of freight, thus leading in 2050 to significantly greater number of passengers (7000–10000 billion passenger-kilometres, in different scenarios) than freight (2300–2800 billion ton-kilometres) in transport provided by diesel. This brings a shift away from diesel-based public transport important. Thus, under the S3 scenario, shifts in the transport sector to tighter emission standards for vehicles and a greater share of CNG in public transport, as well as in the power sector, to non-fossil-power generation reduce NO_*x*_ emissions. Owing to the large shift away from fossil power, the use of selective catalytic reduction (SCR) technology for NO_*x*_ control is not considered. A non-negligible, approximately 20 %, share is from residential, agricultural field burning and brick production sectors, which is reduced in magnitude by the adoption of mitigation, based largely on cleaner combustion technologies. Emissions of NMVOCs increase in 2050 to 16.3 Mt yr^−1^ under the REF scenario, but decrease to about 3.8 Mt yr^−1^ under S3 (Fig. 3g, h, i). In the S3 scenario, mitigation in residential, transport and open burning emissions offsets more than two-thirds of present-day NMVOC emissions. Industrial emissions of NMVOCs, arising primarily from solvent use, are almost constant at 2 Mt yr^−1^ across scenarios, providing further potential for mitigation. However, a shift to public transport based on heavy-duty CNG vehicles drives the increase in NMVOC emissions from the transport sector, from their significantly larger emissions factors, compared to those of heavy duty diesel. Therefore, alternate modes and technologies in the transport sector need further attention.

Anthropogenic dust (Philip et al., 2017), defined here as mineral constituents of pollution particles, including coal fly ash and mineral matter in waste burning and biomass burning emissions, contributes about 30 % of Indian PM_2.5_ emissions in the base year 2015 i.e. about ~ 3 Mt yr^−1^. In future scenarios REF and S2, respectively, anthropogenic dust contributes 6.0 and 4.6 Mt yr^−1^ in 2030 and 12.0 and 6.8 Mt yr^−1^ in 2050, coming primarily (60–85 %) from coal fly ash, with the others coming from fugitive on-road dust and waste burning. In the highest control S3 scenario, anthropogenic dust emissions were reduced to about 1.8 Mt yr^−1^ in both 2030 and 2050. This results from the assumed significant shift to 80–85 % non-coal thermal power generation, leading to large reductions in coal fly ash emissions. Thus, in the S3 scenario anthropogenic dust emissions arise largely from on-road fugitive dust and waste burning (over 50 %), with a lower contribution from coal fly ash (35–40 %).

Emission datasets for India in global emission inventories have been developed either through a combination of regional inventories for specific base years (Janssens-Maenhout et al., 2015) or using integrated assessment models, e.g. the GAINS model (Amann et al., 2011), to generate scenarios of air pollutants (Klimont et al., 2009, 2017, 2018; Purohit et al., 2010; Stohl et al., 2015). Indian emissions for 2008 and 2010 under the HTAP_v2 framework (Janssens-Maenhout et al., 2015) originate from the MIX inventory (M. Li et al., 2017), based on earlier Asia inventories like INTEX-B (Lu et al., 2011; Lu and Streets, 2012) and REAS (Kurokawa et al., 2013). Inconsistencies are reported from merging datasets, calculating different pollutants using differing assumptions (M. Li et al., 2017). The datasets do not include some important regional emission sources like the open burning of agricultural residue (Janssens-Maenhout et al., 2015). Recent global emissions from ECLIPSE V5 (Stohl et al., 2015; http://www.iiasa.ac.at/web/home/research/researchPrograms/air/ECLIPSEv5.html, last access: 1 December 2017), driven by HTAP objectives to improve representation of aerosols emissions in IAMs (Keating, 2015), were reported to have problems over India, including underestimation of BC and trace gas magnitudes and inaccuracies in spatial distribution (Stohl et al., 2015). The present dataset overcomes some of these limitations, using consistent assumptions to calculate a number of pollutants, including all sectors in global inventories, as well as agricultural residue burning emissions and industrial process emissions, while providing for finer spatial resolution using district-level data and more relevant spatial proxies. Emission magnitudes of PM_2.5_ and precursors in present inventory are in good agreement with those in ECLIPSE V5a for 2010; however, those of precursor gases are somewhat lower (about 30 %) than those in HTAP_v2 (2010) and REAS 2.1 (2008) (Sect. S2.6 in the Supplement).

Future emissions of particulate matter (PM_2.5_ and constituents, BC and OC) and precursor gases (SO_2_, NO_*x*_ and NMVOC) estimated here were compared with the more recent sets of scenarios developed with the GAINS model in projects addressing global air pollution trajectories until 2050, i.e. the “Current Legislation scenario” (CLE) of ECLIPSE V5a (Klimont et al., 2017, 2018) and the “New Policies Scenario” (NPS) of World Energy Outlook (IEA, 2016). These scenarios rely on different energy projections; the Energy Technology Perspective study (IEA, 2012) was used in ECLIPSE V5a and World Energy Outlook 2016 in the IEA study. Furthermore, the assumptions about air pollution legislation vary in the IEA study within the “New Policies Scenario”, which includes the recently adopted, announced and planned policies, even when implementation measures were yet fully defined. In general, lower emissions in GAINS-WEO2016-NPS (IEA, 2016) are attributed to the successful implementation of new emission regulations in power and transport sectors, decreased use of biomass fuel in residential sector and phase-out of kerosene lamps. We compare S2 and S3 scenarios in the present study to the baseline scenarios from the above studies (shown in Figs. 2 and 3).

For SO_2_ and NO_*x*_, emission trajectories in the S2 scenario are similar to those in ECLIPSE V5a – CLE – while emissions in the S3 scenario resemble those in GAINS-WEO2016 – NPS – in which newly proposed SO_2_ and NO_*x*_ regulations for thermal power plants and implementation of BS-VI in transportation are included. In fact, the absolute level of emissions estimated for 2015 is also comparable to this study (Fig. 3a, d), though GAINS estimates are slightly higher for SO_2_ and lower for NO_*x*_, owing primarily to differences in emission factors for coal power plants. Bottom-up estimates of SO_2_ emissions from our inventory (Pandey et al., 2014; Sadavarte and Venkataraman, 2014) are consistent with the recent estimates from the satellite-based study (C. Li et al., 2017) from 2005 to 2016, both showing a steady growth. Present-day emissions of SO_2_ (8.1 Mt yr^−1^) are at the lower end of the range of 8.5–11.3 Mt yr^−1^suggested by C. Li et al. (2017). Large future increases in SO_2_ emissions, estimated here in the REF and S2 scenarios, are consistent with the findings of C. Li et al. (2017).

For particulate matter species, the GAINS model estimates lower 2015 emissions, mostly because of the differences for residential use of biomass as well as emissions from open burning. However, considering the uncertainties associated with the quantification of biomass use and emission factors (e.g. Bond et al., 2004; Klimont et al., 2009, 2017; Venkataraman et al., 2010) the differences are acceptable. The future evolution of emissions of BC and OC shows similar features among the studies with S2 comparable to ECLIPSE V5a-CLE and S3 to GAINS-WEO2016-NPS; however the S3 scenario shows a much stronger reduction due to faster phase-out of kerosene for lighting and stronger reduction of biomass used for cooking. The latter feature is especially visible for emissions of OC (Fig. 2d, g). For total PM_2.5_ (Fig. 2a) scenarios developed with the GAINS model do not show a very large difference and fall short of the reductions achieved in the S3 case, in which significant mitigation reduction is not achieved in the residential sector or in the power sector and industry, which in GAINS are either already controlled in the baseline (power sector) or continue to grow, the industrial processes offsetting the benefits of reduction in other sectors.

Emissions of NMVOCs (Fig. 3g) monotonically increase in ECLIPSE V5a-CLE, becoming higher than those in S2 by 2030, which, however, mimic those in GAINS-WEO2016-NPS through to 2050. While there is also a fairly large difference in estimate for the base year (mostly due to residential combustion of biomass, open burning and solvent use sector), obviously the assumptions about the future policies are different as both the ECLIPSE V5a and IEA studies include more conservative assumptions about the reduction of biomass use and eradication of open burning practices, while at the same time they included continued growth in industrial emissions, i.e. solvent applications. Further analysis of differences between the S2 scenario and the ECLIPSE V5a-CLE and GAINS-WEO2016-NPS is shown in the Supplement (Fig. S5).

Further, the emission projections were also compared with emissions estimated in the four representative concentration pathway (RCP) scenarios adopted by the IPCC as a common basis for modelling future climate change (Fujino et al., 2006; Clarke et al., 2007; van Vuuren et al., 2007; Riahi et al., 2007; Hijioka et al., 2008). The RCP scenarios were designed to represent a range of possible future climate outcomes in terms of radiative forcing watts per square metre (W m^−2^) values (2.6, 4.5, 6.0 and 8.5) in 2100 relative to pre-industrial levels. Overall, Indian emissions of SO_2_, NO_*x*_ and BC estimated here in the REF and S2 scenarios, which do not apply stringent controls, were 2 to 3 times higher than the largest emissions estimated in the RCP8.5 scenario in 2030 and 2050, as a result of differences in assumptions made or in the list of sources included (Table S9 in the Supplement). All RCP scenarios considered principally one type of air pollution trajectory, assuming that air pollutant emissions will be successfully reduced with economic growth. Consequently, in the longer term the range of outcomes is fairly similar among RCPs (Amann et al., 2013; Rao et al., 2017). Emissions of these species in the S3 scenario, with the most stringent controls, were in agreement with either RCP8.5 or RCP4.5 scenario emissions. Emissions of OC in the REF and S2 scenarios and of NMVOCs in the S2 and S3 scenarios were in agreement with the ranges estimated in the RCP4.5 and RCP8.5 scenarios. Emissions of SO_2_ estimated here for the highest-control scenario, S3, agreed with those from RCP4.5 in 2030 and RCP8.5 in 2050, due to similar assumptions of over 80 % non-coal electricity generation. However, the S2 and REF scenarios estimated much larger emissions. Further details are presented in Sect. S2.6 in the Supplement.

## 3 Model simulations and evaluation

The emissions were input in the GEOS-Chem model (http://www.geos-chem.org, last access: 1 December 2017) to calculate pollutant concentration fields in space and time. The GEOS-Chem model has been previously applied to study PM_2.5_ over India (e.g. Boys et al., 2014; Kharol et al., 2013; Philip et al., 2014a; M. Li et al., 2017), including relating satellite observations of aerosol optical depth to ground-level PM_2.5_ for the GBD assessment (Brauer et al., 2012, 2016; van Donkelaar et al., 2010, 2015, 2016). The simulations undertaken in this work represent one of the best efforts to date as they include the full Indian spatial domain and fine spatial resolution, as well as global-scale atmospheric processes.

In addition to the emissions described in Sect. 2.2.2, other emissions such as open burning, except agricultural residue burning which includes forest fires, were derived from the global GFED-4s database (Akagi et al., 2011; Andreae and Merlet, 2001; Giglio et al., 2013; Randerson et al., 2012; van der Werf et al., 2010). In addition to the species in this inventory, ammonia or NH_3_ emissions, important for calculating secondary particulate matter, were taken from the MIX emission inventory (M. Li et al., 2017; http://meicmodel.org/dataset-mix.html, last access: 1 December 2017). Emissions of NH_3_ arise primarily from sources like animal husbandry, not addressed in the present inventory. Therefore, they are taken from M. Li et al. (2017). Owing to large uncertainties in future emissions, these were kept the same in future scenarios as for 2015. Emission magnitudes of NH_3_ could affect secondary nitrate, which typically contributes to less than 5 % of PM_2.5_ mass (Fig. 5a, c; Kumar and Sunder Raman, 2016; Ram and Sarin, 2011; Rastogi et al., 2016), thus not influencing overall results in any significant manner. The model focuses on the temporal and spatial evolution of aerosols and gaseous compounds using meteorological datasets, emission inventories and equations that represent the physics and chemistry of the atmosphere. Version 10.01 is used here. Total NMVOC emissions from India were taken from Sarkar et al. (2016). The GEOS-Chem model speciation (Table S10, Supplement), into eight species, was applied for further input to the photochemical module. The simulation of PM_2.5_ includes the sulfate–nitrate–ammonium–water system (Park et al., 2004), primary (Park et al., 2003) and secondary (Henze and Seinfeld, 2006; Henze et al., 2008; Liao et al., 2007; Pye et al., 2010) carbonaceous aerosols, mineral dust (Fairlie et al., 2007), and sea salt (Alexander et al., 2005). The GEOS-Chem model has fully coupled ozone–NO_*x*_–hydrocarbon chemistry and aerosols including sulfate $\left({\text{SO}}_{4}^{2-}\right)$, nitrate $\left({\text{NO}}_{3}^{-}\right)$, ammonium $\left({\text{NH}}_{4}^{+}\right)$ (Park et al., 2004; Pye et al., 2009), organic carbon (OC) and black carbon (BC) (Park et al., 2003), sea salt (Alexander et al., 2005) and mineral dust (Fairlie et al., 2007). For these simulations we also included the ${\text{SO}}_{4}^{2-}$ module introduced by Wang et al. (2014). Partitioning of nitric acid (HNO_3_) and ammonia between the gas and aerosol phases is calculated using ISORROPIA II (Fountoukis and Nenes, 2007). Secondary organic aerosol formation includes the oxidation of isoprene (Henze and Seinfeld, 2006), monoterpenes and other reactive volatile organic compounds (Liao et al., 2007) and aromatics (Henze et al., 2008).

The South Asia nested version of GEOS-Chem used here was developed by Sreelekha Chaliyakunnel and Dylan Millet (both of the University of Minnesota) to cover the area from 55 to 105° E and from 0° S to 40° N and to resolve the domain of South Asia at a resolution of 0.5° × 0.67° (approximately 56×74 km at equator) with dynamic boundary conditions using meteorological fields from the NASA Goddard Earth Observation System (GEOS-5). The boundary fields are provided by the global GEOS-Chem simulation, with a resolution of 4° latitude and 5° longitude (approximately 445×553 km at equator), which are updated every 3 h. We have corrected the nighttime mixing depths that are too shallow and the overproduction of HNO_3_ in the model following Heald et al. (2012) and Walker et al. (2012). We applied the organic mass to organic carbon ratio in accordance with findings from Philip et al. (2014b). A relative humidity of 50% was used to represent simulated PM_2.5_ measurements in India. South Asia nested meteorological fields were not yet available post-2012 due to a change in the GEOS assimilation system in 2013. Therefore, we conducted standard simulations to test meteorology from the years 2010 to 2012.We chose the year 2012 as our meteorology year, as the simulation results using this year best represented the mean PM_2.5_ concentration from 2010 to 2012. A 3-month initialization period was used to remove the effects of initial conditions.

To estimate the impacts of individual sources, simulations were made using total emissions from all sources, along with sensitivity simulations (Table 1) for major sources. Sources included in the standard simulation, however, that were not separately addressed in sensitivity simulations, termed “other”, include residential lighting with traditional kerosene lamps and informal industry (food and agro-product processing). Primary particulate matter is largely composed of carbonaceous constituents (black carbon and organic matter) and mineral matter. Mineral matter from combustion and industry, calculated as the difference between emitted PM_2.5_ mass and the sum of black carbon and organic matter, each calculated from respective emission factors and lumped along with urban fugitive dust and evaluated in a previous study (Philip et al., 2017), is termed anthropogenic fugitive dust or ADST. For sensitivity simulations, the total coal-related emissions, industrial coal-related emissions and emissions from other major sectors are each removed from the inventory in each scenario. The global and nested grid models of GEOS-Chem were then run in sequence using the new inventories. These sensitivity simulation results therefore depict the ambient PM_2.5_ concentrations with each emission sector shut off. The differences of the standard and sensitivity simulations were analysed to produce contributions of the individual sectors to ambient PM_2.5_ concentrations. By comparing the difference in simulated ambient concentrations between the standard and sensitivity simulations, we therefore consider in our analyses the complex non-linear relationships between emissions and ambient concentrations and the non-linear atmospheric chemistry affecting particle formation.

**Figure 4.**
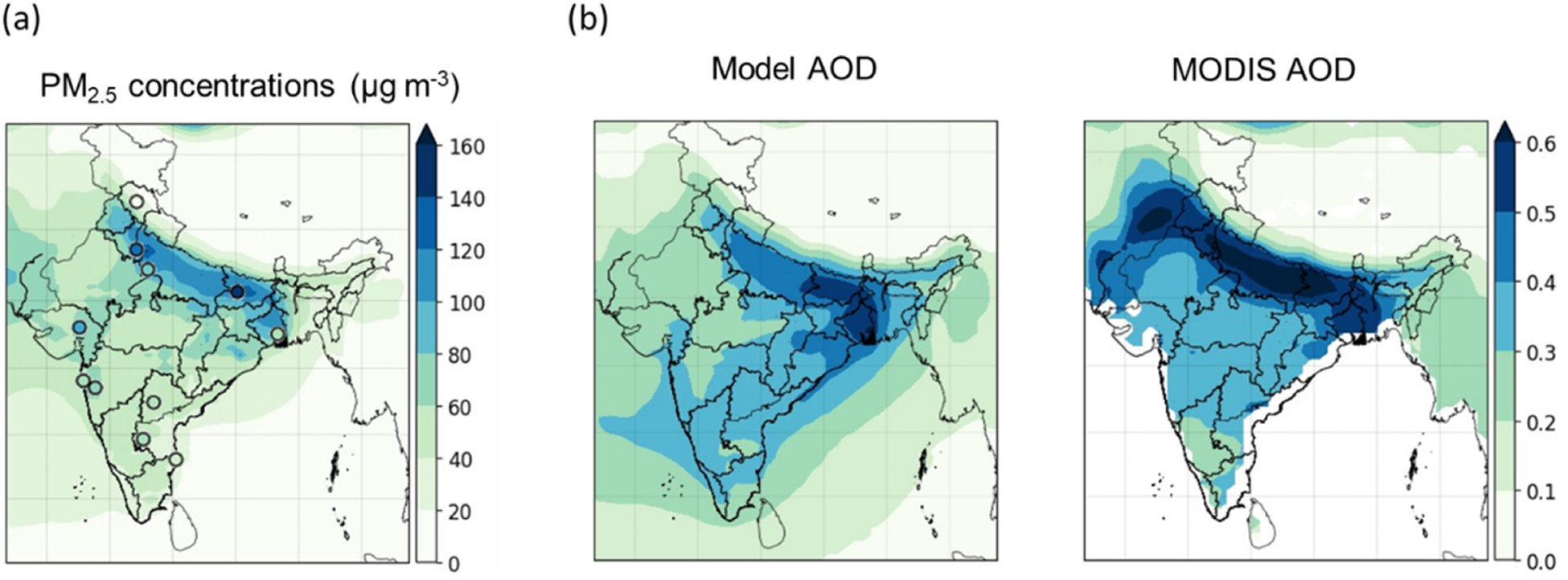
Model evaluation by **(a)** comparison of simulated annual mean PM_2.5_ concentrations with in situ observations (circles represent observations) and **(b)** comparison of modelled annual mean AOD over India with observations from MODIS.

**Figure 5.**
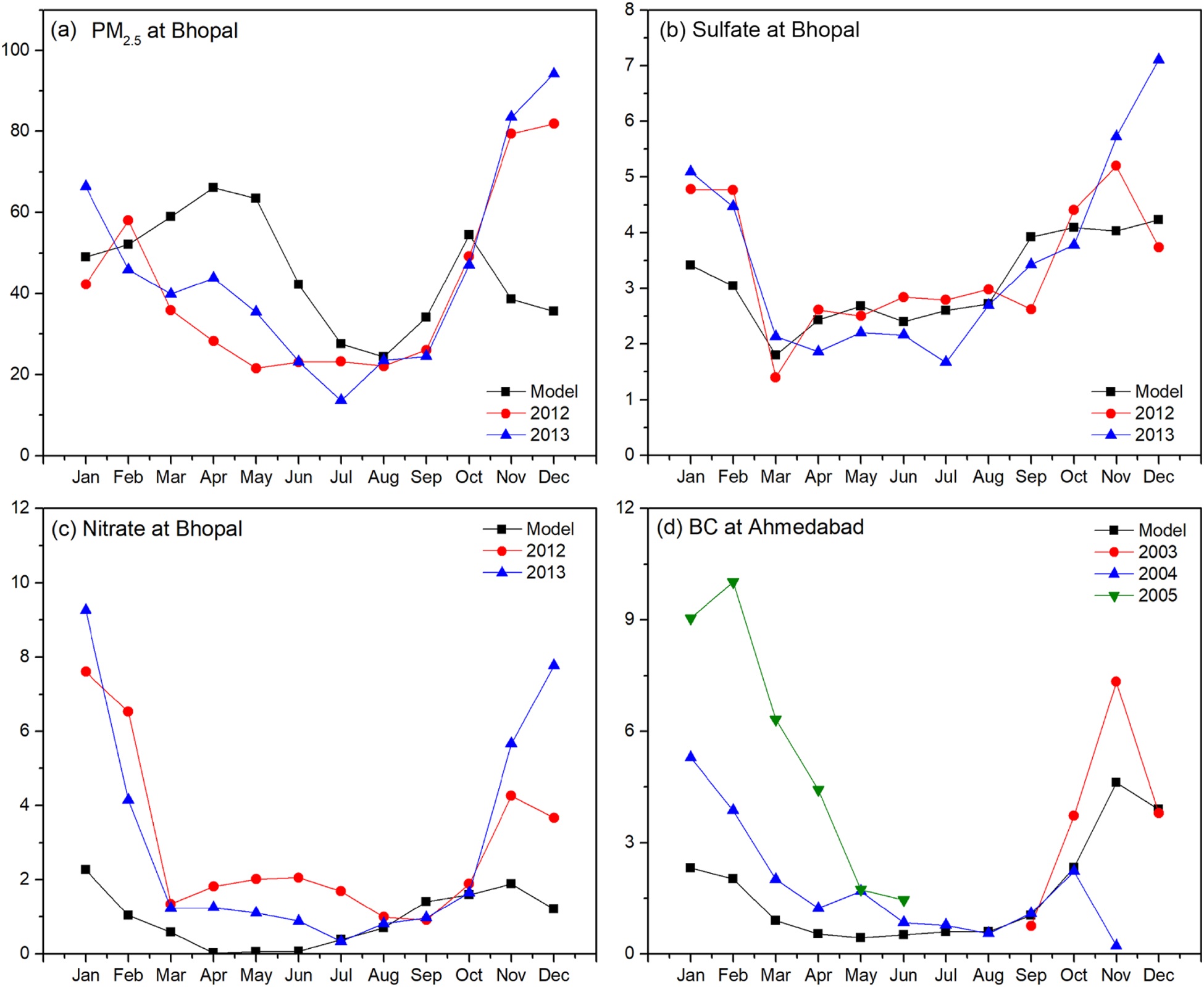
Evaluation of model performance (NMB) in capturing seasonal variation in chemical species concentrations at two sites in India.

The GEOS-Chem simulations made here include those for primary aerosol emissions (secondary sulfate, nitrate and ammonium) and secondary organic aerosol, going beyond previous simulations made on regional scales over India (e.g. Sadavarte et al., 2016), which were limited to secondary sulfate and a smaller list of sources in the emissions inventory, addressing only a few months in the year. Model-predicted concentrations of PM_2.5_ (Fig. 4) and its chemical constituents (Fig. 5) were evaluated against available PM_2.5_ measurements, satellite observations of columnar aerosol optical depth (AOD) and available monthly chemical composition measurements (Kumar and Sunder Raman, 2016; Ramachandran and Kedia, 2010; Ramachandran and Rajesh, 2007). Model performance was evaluated through normalized mean bias (NMB) (Eq. 1) for pairs of model-predicted concentrations (*M*) and corresponding observed concentrations (*O*), at given locations and for the same averaging period:

(1) $$\text{normalized}\;\text{mean}\;\text{bias}\;=\frac{\sum_{1}^{n}\left(M-O\right)}{\sum_{1}^{n}\left(O\right)}.$$

The evaluation of the seasonal cycle of simulated PM_2.5_ is inhibited by the paucity of measurements. Evaluation of the PM_2.5_ seasonal variation reveals an overall general consistency between the simulation and observations. However, some of the largest concentrations, e.g. at Delhi (28.6° N, 77.1° E) and Kanpur (26.4° N, 80.3° E), were somewhat underestimated. The model captures AOD distribution over large parts of India, compared to measurements from MODIS (Fig. 4b; NMB of −33 %), but appears to show an underestimation in the northwest, implying underestimation in modelled windblown dust emissions in the Thar Desert. However, the evaluation may be interpreted with caution, from differences arising from sensor (e.g. MODIS and MISR) variability in the AOD product both spatially and temporally over India (Baraskar et al., 2016), as well as a lack of coincident sampling of model with satellite observations.

Evaluation was also explored against monthly mean chemical composition measurements (Fig. 5) at a regional background site (Bhopal, 23.2° N, 77.4° E; Fig. 5a, b, c; PM_2.5_, sulfate, nitrate; methods described in Kumar and Sunder Raman, 2016) and a western urban site (Ahmedabad, 23.0° N, 72.5° E; Fig. 5d, BC; aethalometer measurements in Ramachandran and Rajesh, 2007). The simulation captures monthly PM_2.5_ and species mean concentrations satisfactorily during non-winter months at the two sites but with some underestimation in the winter months. While sensitivity simulations for nitrate (not shown) increased nitrate concentrations in northern India, they were largely unchanged in central India, evident in the underestimation of nitrate (NMB of −68 %) at Bhopal. The spatial distribution of particulate species (not shown) reflects the interplay of emission density distributions with transport processes, with sulfate showing a predominance in central India and to the east where there is a prevalence of thermal power generation, but BC and organic matter showing a predominance in northern India, where there is a prevalence of traditional biomass fuelled residential energy technologies. The findings here are broadly consistent with earlier work (Sadavarte et al., 2016), which showed large surface concentrations of sulfate, organic carbon and dust over northern India.

As discussed earlier, NMVOC emissions from India were taken from a recent technology-linked inventory, deployed in WRF-CAMx and evaluated with satellite and in situ observations (Sarkar et al., 2016). However, uncertainties still remain to be addressed in the calculation of secondary PM_2.5_ constituents, especially secondary organic aerosols, whose precursor NMVOC emissions in developing countries are still uncertain from a lack of speciation measurements under combustion conditions (Roden et al., 2006; Martinsson et al., 2015) typically encountered in traditional technologies in residential cooking and heating as well as in informal industry, including brick production. Recent studies (Stockwell et al., 2016) attempted to fill this gap. Such findings must be incorporated into future emission inventory evaluations in order to further refine regional PM_2.5_ calculations. While the present study did include calculation of both primary and secondary organic matter as constituents of PM_2.5_, a detailed study of the sources and fate of total or secondary organic aerosol over the Indian region is beyond the scope of this work. Direct comparison of spatially averaged model output with satellite products or in situ measurements typically incorporates significant uncertainty. A broad evaluation was undertaken here, without a match of model output to specific sampling time or satellite overpass time. Thus, some differences would arise from modelled meteorology not faithfully representing actual meteorological conditions during the measurement period. With these caveats, we acknowledge the need for coherent measurement campaigns to map concentrations of both PM_2.5_ and its chemical constituents over India, to improve model evaluation and future air quality management.

## 4 Simulated PM_2.5_ concentrations by state and sector

### 4.1 Present-day and future PM_2.5_ concentrations at national and state levels

**Figure 6.**
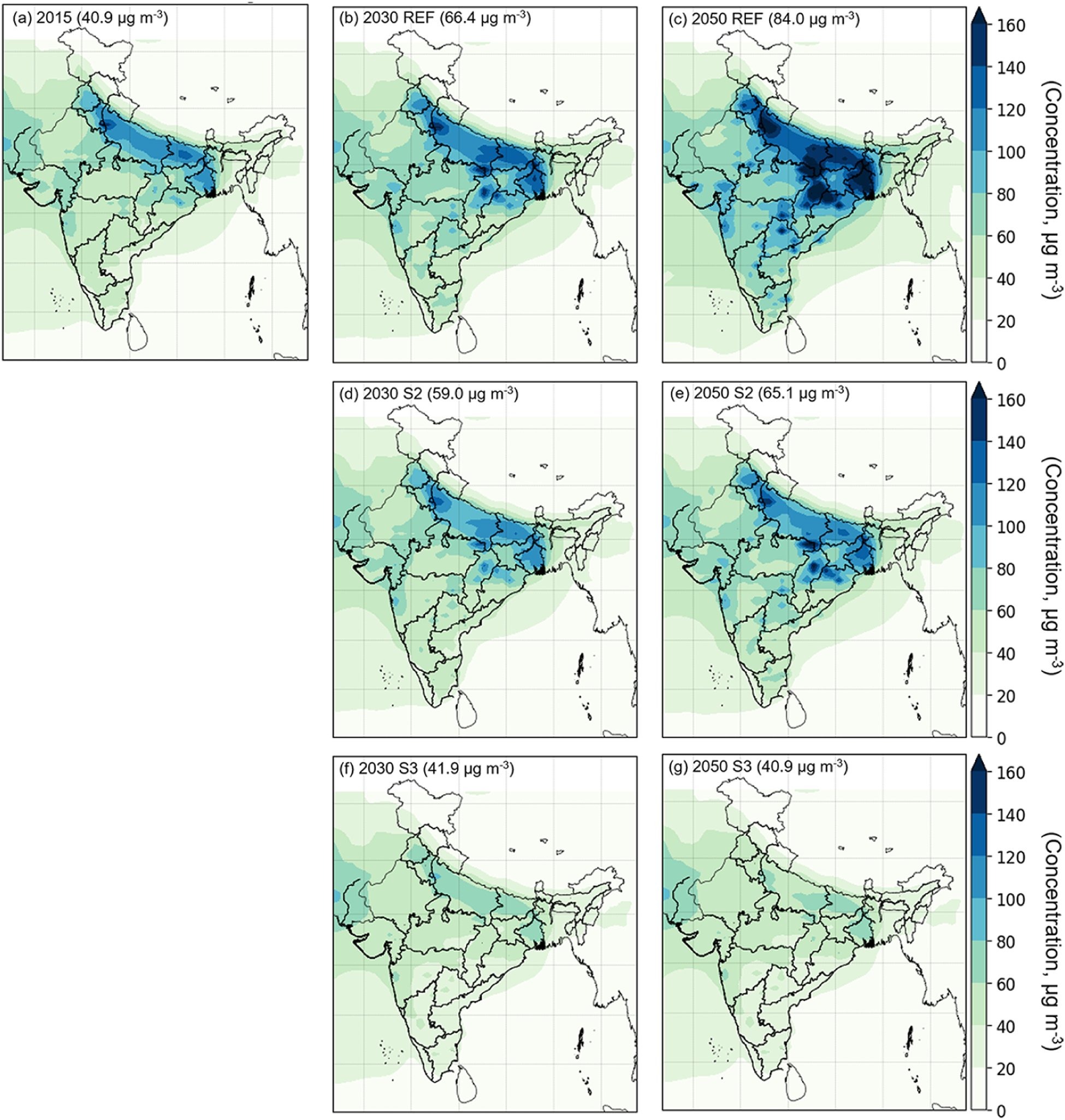
Simulated PM_2.5_ concentration in **(a)** 2015, **(b)** 2030 REF, **(c)** 2050 REF, **(d)** 2030 S2, **(e)** 2050 S2, **(f)** 2030 S3 and **(g)** 2050 S3. (Values in the parentheses represent area-weighted average PM_2.5_ concentration for India.)

We find that ambient PM_2.5_ pollution is a pan-India problem with a regional character. Figure 6a–g show the simulated total ambient PM_2.5_ concentrations for 2015 and in each future scenario (REF, S2, and S3) for 2030 and 2050 to illustrate the different spatial patterns under each scenario. The figure displays mean PM_2.5_ concentration at a grid level, with area-weighted mean values shown in parentheses. Figure 6a shows the simulated annual mean PM_2.5_ concentrations in 2015. It illustrates that the ambient PM_2.5_ concentration has a clear regional distribution, with high values in northern India. High PM_2.5_ concentrations in northern India can be attributed both to higher local emissions, especially of organic carbon, and to synoptic transport patterns, leading to confinement of regional emissions of particulate matter and precursor gases in the northern plains (e.g. Sadavarte et al., 2016), manifested in high concentrations of secondary particulate sulfate and dust. In most parts of India values exceed the Indian National Ambient Air Quality Standard (CPCB, 2009) of 40 μg m^−3^ for annual mean PM_2.5_, with values as high as 140 μg m^−3^ in northern India. Large regions of north, eastern and western India exhibit high PM_2.5_ concentrations, which are not just limited to specific urban centres or megacities examined in earlier studies (Jain and Khare, 2008; Guttikunda and Jawahar, 2012; Sharma and Maloo, 2005).

Simulations with the REF scenario emissions (Fig. 6b, c), show significant increases in annual mean PM_2.5_ concentrations all over India, preserving a similar elevated spatial pattern in the northern and northeastern regions, resulting from significant increases in emissions of primary PM_2.5_ and its precursors from their 2015 values. The REF scenario also results in significant increases, over 2015 levels, in area-averaged PM_2.5_ concentrations over India in 2030 (62.3.7 %) and 2050 (105.4 %) (shown in Fig. 6a, b, c). The largest future PM_2.5_ concentration values approach 164.1 μg m^−3^ in 2030 and 323.3 μg m^−3^ in 2050 in the REF scenario. Under the S2 scenario, simulated concentrations are projected to improve relative to REF, following similar spatial patterns, with the northern and northeastern regions remaining as the most polluted areas. However, there is no appreciable change in nationally averaged PM_2.5_ concentrations in 2030, while there is even a modest increase in 2050. This implies that energy-use and emission evolution, under both current regulation (REF) and that which is promulgated or proposed (S2), are not expected to yield significant improvements in future air quality. Under the S3 scenario, a total shift away from traditional biomass technologies and a very large shift (80–85 %) to non-fossil electricity generation (S3 scenario) controls the increase in overall PM_2.5_ concentrations and leads to a reduction in spatial variability within India. Under this scenario, the PM_2.5_ concentrations are found to stabilize at 2015 levels, without any significant increase in 2030 and 2050 (Fig. 6a, f, g). The mean population-weighted PM_2.5_ concentrations for 2015 and future scenarios for India are shown in Fig. S7 in the Supplement. The uncertainty represented by the bars is based on uncertainty in the GBD estimates of ambient PM_2.5_ concentrations. It is estimated by sampling 1000 draws of a distribution for each grid cell based on the model output mean and standard deviation (GBD MAPS Working Group, 2018).

**Figure 7.**
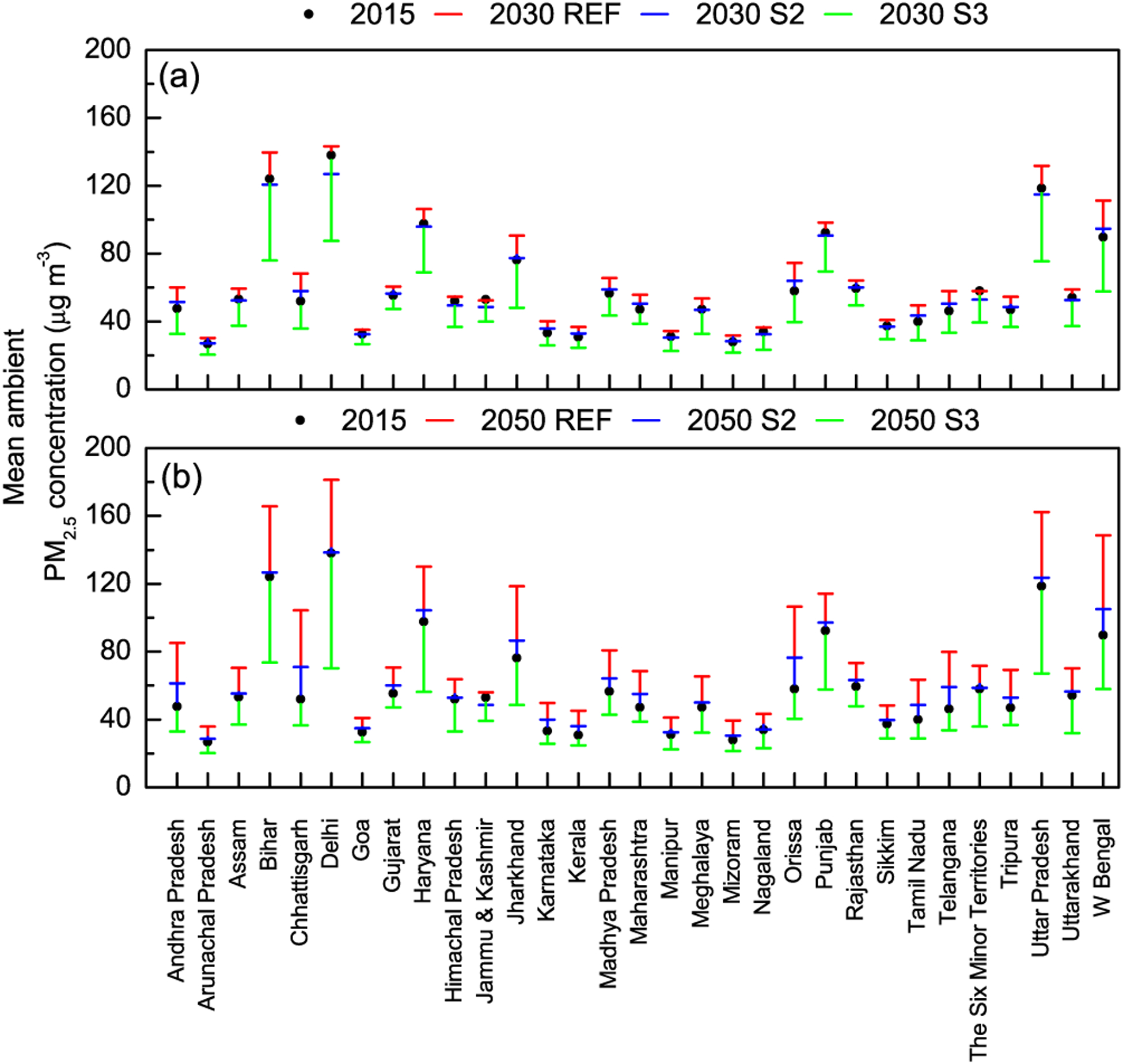
Population-weighted mean ambient PM_2.5_ concentrations by state for **(a)** 2015 and 2030 (REF, S2 and S3) and **(b)** 2015 and 2050 (REF, S2 and S3).

We further examine which increases or decreases in PM_2.5_ concentrations occur at the state level. India is organized administratively into 29 states and 7 union territories; therefore evaluating state-level PM_2.5_ concentrations provides information useful at the regulatory level of state pollution control boards (Air (Prevention and Control of Pollution) Act, 1981). At the state level, changes in future PM_2.5_ concentrations, from their 2015 levels, were evaluated under the three scenarios (Fig. 7a, b). Simulated PM_2.5_ concentrations from the model are weighted by population for each state. This is calculated by multiplying the concentration in each grid cell (0.1 × 0.1°) by the population, summing this quantity for all grid cells that lie within a state and then dividing it by the total population in each state. Under present-day emissions of 2015, populations-weighted mean concentrations in most states were above the national PM_2.5_ standard, except for Nagaland, Karnataka, Goa, Manipur, Mizoram, Kerala, Sikkim and Arunachal Pradesh. In 2030, under the REF scenario, significant increases from 2015 levels were projected in PM_2.5_ in Bihar, Haryana, Jharkhand, Odisha and Uttar Pradesh, while under the S2 scenario, increases were projected in states such as Chhattisgarh, Odisha and West Bengal. This implies worsening future air quality in these locations under assumptions of current and promulgated future regulations. However, under the S3 emission scenario, which includes control beyond currently promulgated regulations, significant decreases in PM_2.5_ in 2030 were projected, with 20 states and 6 union territories reaching population-weighted mean concentrations below the national ambient air-quality standard, with the largest reductions in Andhra Pradesh, Chhattisgarh, Himachal Pradesh and Odisha. However, 10 states (including Delhi) were projected to continue to have population-weighted mean concentrations above the national PM_2.5_ standard in 2030, even under the lowest emission scenario in this study.

A similar picture was seen in 2050 as well, with very significant increases under the REF scenario in all states, leading to extreme PM_2.5_ concentrations between 100 and 200 μg m^−3^ in over 10 states (including Bihar, Chhattisgarh, Delhi, Haryana, Jharkhand, Punjab, Uttar Pradesh and West Bengal). Under S2 scenario emissions there was either no appreciable change or a modest increase in projected PM_2.5_ levels (in states including Andhra Pradesh, Chhattisgarh, Orissa, Telangana and West Bengal). Again, only under S3 scenario emissions was there a significant reduction in projected future PM_2.5_ levels, with the same 20 states and 6 union territories falling below the national PM_2.5_ standard; however, the same 10 states (including Delhi) still continue to experience population-weighted mean concentrations higher than the standard.

### 4.2 Simulated source contributions to present-day and future PM_2.5_ concentrations at national and state levels

**Figure 8.**
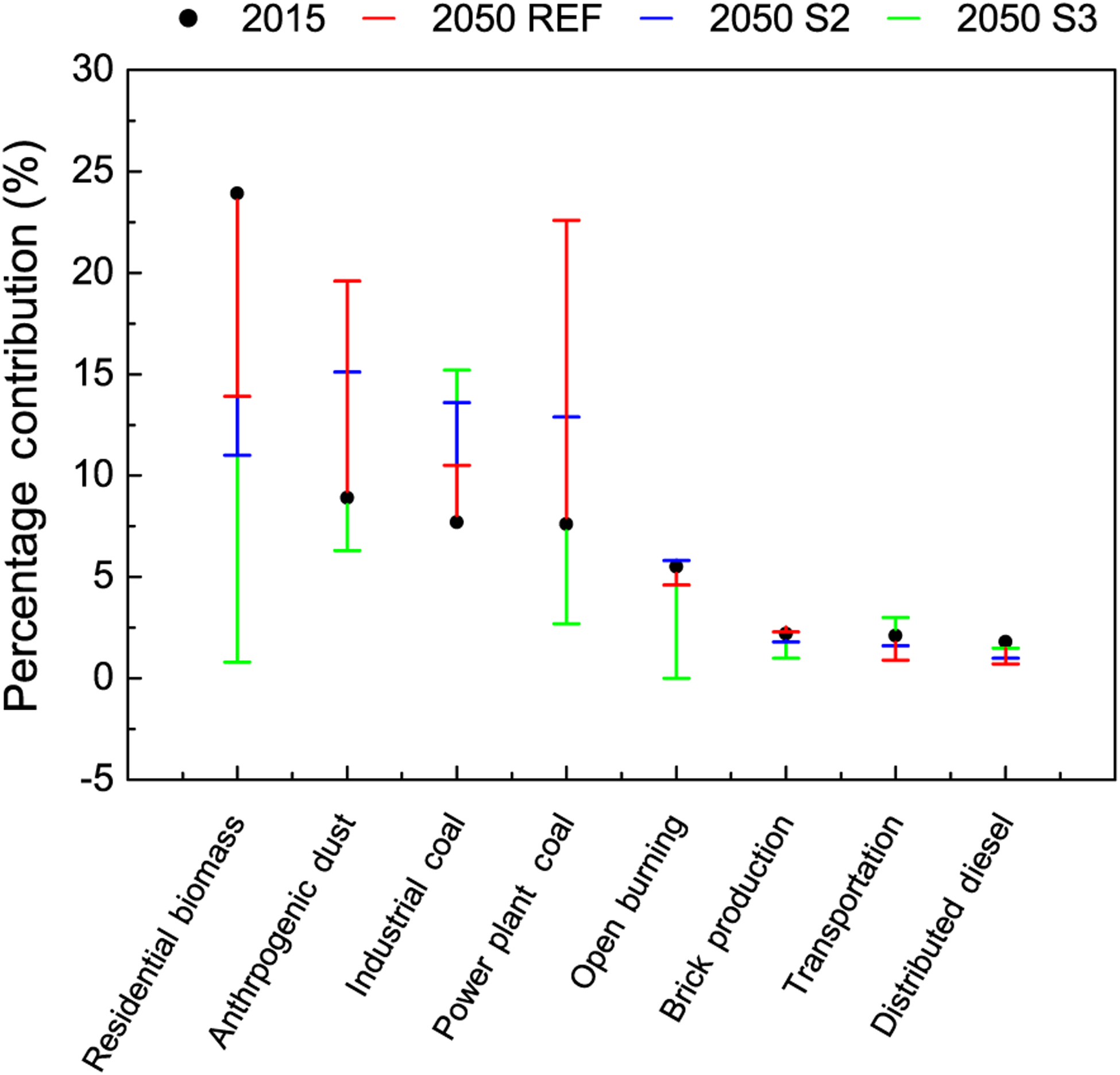
Percentage contribution to ambient PM_2.5_ attributable to different sources in 2015 and 2050 for all three scenarios.

**Figure 9.**
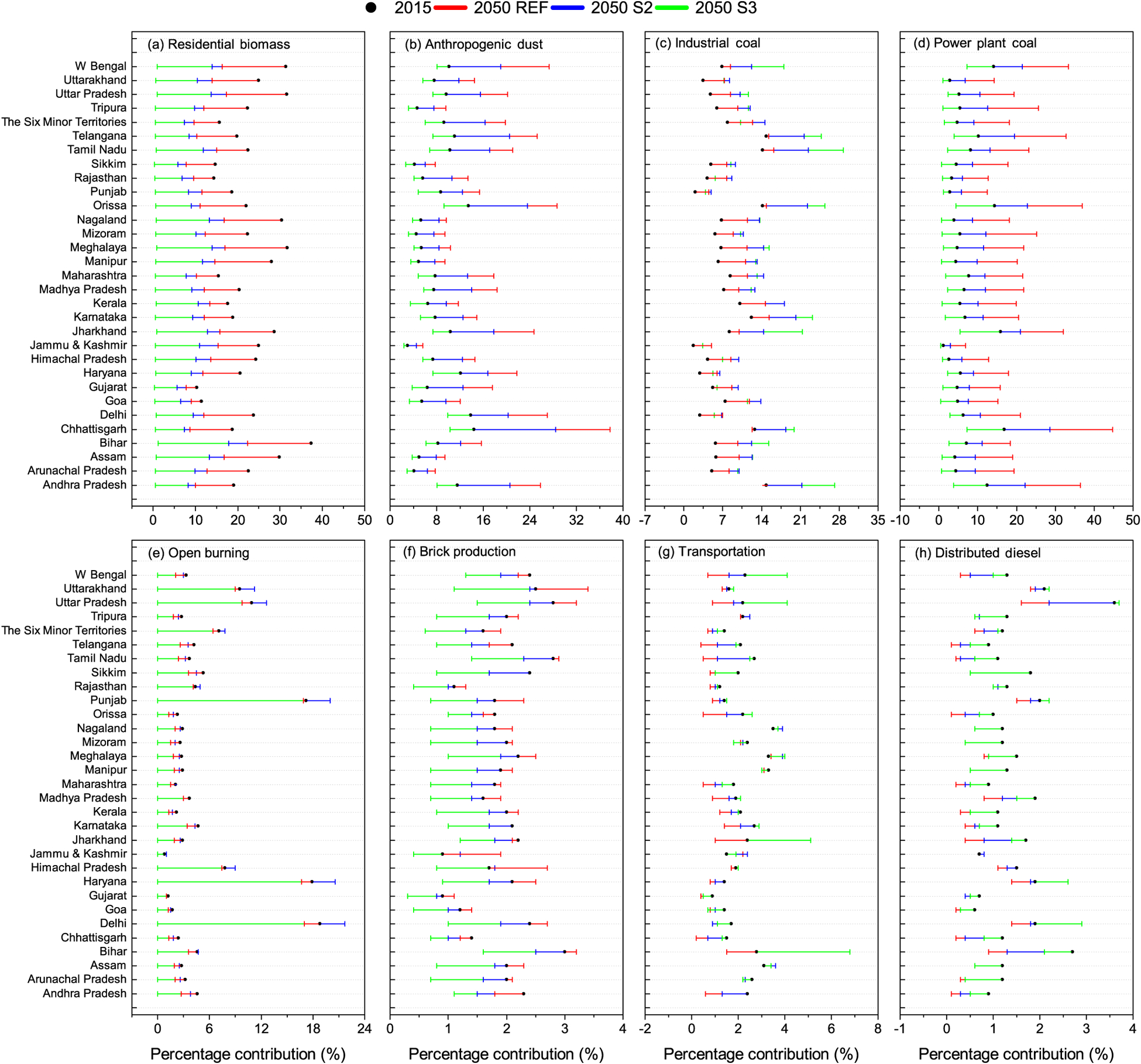
Percentage contribution of **(a)** residential biomass, **(b)** anthropogenic dust, **(c)** industrial coal, **(d)** power plant coal, **(e)** open burning, **(f)** brick production, **(g)** transportation and **(h)** distributed diesel attributable to ambient PM_2.5_ concentration by state (2015–2050).

The simulated change in sectoral contribution to population-weighted PM_2.5_ concentrations is evaluated both at national (Fig. 8) and at the state level (Fig. 9). The figures show the simulated percentage contributions to PM_2.5_ from residential biomass, anthropogenic dust, power plant coal, industry coal, open burning (agricultural), transportation, fired-brick production and distributed diesel sectors. It should be noted that the sum of contributions from all subsectors does not add up to the simulated ambient concentration from all emission sources. This results from the non-linearity in the relationship between emissions and ambient concentrations. Non-linearity is related to atmospheric motion and to atmospheric reactions which are highly non-linear both in space and time, which lead to formation of secondary PM_2.5_ constituents, like sulfate, nitrate and organic carbon. Further, estimation of the fractional contribution from each sector is based on a difference between pairs of simulations, one based on all sources and a sensitivity simulation in which that source sector is removed. Since source-sector-based sensitivity simulations were made only for 2015 and 2050 (but not 2030), the figures depict the contribution of the simulated source sectors in 2015 and that from the three scenarios in 2050. Source contributions have to be interpreted with caution, since they are calculated relative to the total of all sources for a particular year and a particular scenario.

In 2015, among source sectors, the single largest contributor to ambient PM_2.5_ was residential biomass fuel use for cooking and heating, followed by anthropogenic dust, industrial and power plant coal burning and the open burning of agricultural residue. Emissions from fired-brick production, transportation and distributed diesel (diesel generator sets) also have some contribution to air pollution. It is noteworthy that outdoor air pollution in present-day India is dominated by residential biomass fuel use, which is primarily known to contribute to a significant burden of disease in India via household air pollution exposures (GBD 2016 Risk Factors Collaborators, 2017). Prior global analyses have also found evidence for the importance of residential biomass fuel use in India (e.g. Verma et al., 2008, 2011; Philip et al., 2014b; Lelieveld et al., 2015; Silva et al., 2016; Lacey et al., 2017). The dominance of residential biomass fuel emissions is an important underlying cause for the regional nature of air pollution in India because of the widely dispersed and distributed nature of this uncontrolled source. Overall, sources related to human activities were found to be responsible for the largest proportion of the present-day population exposure to PM_2.5_ in India. PM_2.5_ concentrations attributable to sources outside India mainly originate from regions to the west of the country; therefore their contributions to the regional background vary considerably by region. Transboundary pollution is highest in the northwestern regions, where it contributes about 15 to 30 % (> 12 μg m^−3^), and it is lowest in the southern part of the country, where the contributions are less than 15 % (4–8 μg m^−3^). About 60 % of India’s mean population-weighted PM_2.5_ concentrations come from anthropogenic source sectors, while the remainder are from “other” sources, windblown dust and extra-regional sources. Leading contributors are residential biomass combustion, power plant and industrial coal combustion and anthropogenic dust (including coal fly ash, fugitive road dust and waste burning). Total dust (windblown and anthropogenic) together contributed 39 %, while transportation, brick production and distributed diesel were other contributors to PM_2.5_.

In 2050, future source contributions are dominated by power plant coal and industrial coal, in both REF and S2 scenarios, followed by residential biomass. In both REF and S2 scenarios (Figs. 2 and 3) expansion in electricity generation and industry overtakes emissions offsets, leading to 1.5–2 and 1.75–3 times emission increases, respectively, in emissions of PM_2.5_ and its precursor gases, through to 2050. The future expansion projected in power plant and industrial coal use, in both these scenarios, exceeds the growth in biomass fuel use in the residential sector, which follows population increases. Future source contributions to emissions of PM_2.5_ and precursor gas emissions are about 60 % from coal burning in electricity generation and industry, while the remainder are from biomass energy use in the residential sector, which is directly reflected in source contributions to ambient PM_2.5_. The power plant coal contribution to PM_2.5_ increases in the REF and S2 scenarios; however, it decreases in the S3 scenario, from assumptions of very high penetration (80–85 %) of non-fossil electricity generation. The industrial coal contribution to PM_2.5_ concentrations increases above 2015 levels in all future scenarios, reflecting expansion in industry and related “process emissions”. This finding suggests that even more stringent measures than those assumed in the scenarios are needed to reduce the influence of industrial coal combustion on ambient pollution levels.

Interestingly, the influence of residential biomass emissions on PM_2.5_ reduces in 2050, even in the REF scenario, from the relative increase in that of industrial coal. In the S2 and S3 scenarios, assumptions of a future shift from residential biomass to cleaner LPG/PNG and advanced low-emission gasifier stoves leads to its decreased contribution to PM_2.5_ concentrations. In the S3 scenario, assumptions of a complete switch away from traditional residential biomass technologies leads to this sector having the lowest influence on PM_2.5_ concentrations (less than 1.8 %). The validity of such assumptions rests upon careful review and effective implementation of national programmes recently launched for the expansion of cleaner residential fuels (Pradhan Mantri Ujjwala Yojana, 2016) as well as sustainable adoption of these low emissions approaches. The influence of anthropogenic dust is projected to increase in REF and S2 scenarios, while a decrease is only observed in the S3 scenario. On the other hand, the influence of total dust is projected to increase in all future scenarios, largely from decreases in the influence of other PM_2.5_ sources. Total and anthropogenic dust concentrations are projected to increase under all scenarios. Dust from anthropogenic activities (anthropogenic dust) is a larger contributor to total dust in REF (47 % of total dust, compared with 23 % in 2015) and S2 (36 % of total dust), while its contributions in S3 (13 %) are low. Overall, in S3, total dust (in this scenario dominated by windblown mineral dust) is the largest contributor to ambient PM_2.5_ as a result of the dramatic reductions in emissions projected for all of the other sectors (including anthropogenic dust) in this ambitious scenario. Further examination is needed of the contribution and amelioration of sources in the “other” category, not simulated separately here, which includes waste burning, urban fugitive dust, residential lighting with kerosene and informal industry related to food and agricultural product processing, which relies on traditional technologies and biomass fuel.

The PM_2.5_ concentration from transportation sources remains low (< 2 μg m^−3^) under all scenarios but does not decrease in the ambitious scenario. This is related both to the lower magnitude of transportation emissions, relative to other sources, as well as the relatively coarse model grid (50 km × 67 km). That the transportation contribution decreases in REF but increases in S3 relative to 2015 reflects competing trends from 2015 to 2050 where emissions per vehicle generally decrease but with an increase in vehicle-kilometres. Specifically, passenger-kilometres increase about 4-fold from 2015 to 2050 but with reductions of 15 to 55 % in primary PM_2.5_ emissions along with increases in transport-related SO_2_ (27 to 73 %) and NO_*x*_ (93 to 121 %) emissions, depending on the scenario. Further, emissions from transportation may be affected by reductions in emissions from other sectors and non-linear atmospheric chemistry (e.g. reductions in other combustion sources leaving more ammonia available to react with transportation combustion products to form secondary PM). Indeed, evaluation of simulation results indicates that the sensitivity of nitrate to transportation sources in scenario S2 is larger than the nitrate sensitivity in the REF scenario. This suggests that increased available ammonia in S2, resulting from reductions in emissions from other sectors, leads to increased particulate ammonium nitrate formation associated with transportation emissions, relative to the REF scenario. Furthermore, for a number of reasons – because we are estimating sectoral contributions to ambient PM_2.5_ based on the fractional contribution from each sector, because transportation is small relative to the other sectors and because the spatial pattern of the fraction of transport emissions does vary from scenario to scenario – it is also possible that the decrease in REF, followed by increases in S2 and S3, is an artefact due to increasing fractional contributions from transport relative to other sectors where the decreases are much more dramatic.

Changes in source contributions to PM_2.5_, between 2015 and 2050, are analysed at state level (Fig. 9), wherein patterns similar to those at the national level are seen. Residential biomass fuel use (Fig. 9a) was the dominant source influencing PM_2.5_ in 2015, on both national and state scales. The trade-off between relative decreases in residential biomass and increases in industrial coal on future PM_2.5_ is seen in the REF, S2 and S3 scenarios, at the state level. In Fig. 9a (residential biomass) note that the red, blue and green lines lie below the black dots, while in Fig. 9c (industrial coal) they all lie above the black dots, and in Fig. 9d (power plant coal) only red and blue lines lie above the black dots. Residential biofuel influence reduces in all scenarios in 2050, reaching between 1 and 2 % at the state level, across all states. Anthropogenic dust (Fig. 9b) shows decreasing influence, while total dust shows increasing influence on PM_2.5_ in the S3 scenario, even at the state level, for reasons discussed above. There is an increase in the influence of industrial coal (Fig. 9c) on PM_2.5_ in all states under all three scenarios because of expansion, for the same grid locations, in industrial production and related “process” emissions, e.g. grinding and milling operations in the cement industry, despite improved technology efficiencies assumed in the industrial sector. Industrial emission increases are highest in Andhra Pradesh, Jharkhand, Karnataka, Odisha and Tamil Nadu. Further refinement of scenarios must be made to include more stringent industrial emission control technologies. The power plant coal (Fig. 9d) influence increases in the REF and S2 scenarios in all states; however the largest increases are seen in Andhra Pradesh, Chhattisgarh, Odisha, West Bengal and Telangana. Under S3 scenario emissions, the power plant coal influence decreases in all states, but it has the largest decreases in the same states as above, indicating that the emissions are influenced by high electricity generation in these states, with uniform assumptions made on the shift to non-fossil generation. However, future PM_2.5_ levels are strongly influenced by industrial and power plant coal use across most states. The influence of open burning (Fig. 9e) appears to change in 2050 under REF and S2 scenarios, not from absolute changes in open burning, but from changes relative to decreases in the influence of other sources. However, under S3 scenario emissions, in which a complete phase-out of open burning is assumed, there are uniform decreases in all states, leaving a negligible influence. The influence of brick production (Fig. 9f) on PM_2.5_ has a negligible increase in the REF scenario at the national level; however, it shows significant increases at the state level from 2015 to 2050 in Bihar, Himachal Pradesh, Punjab, Uttar Pradesh and Uttarakhand, the major brick-producing states. While the influence of brick production decreases in almost all states under the S3 scenario, it still contributes about 2 % in these states through to 2050. The influence of transportation (Fig. 9g) increases significantly under the S3 scenario in a few states like Bihar, Jharkhand, Uttar Pradesh and West Bengal, a likely artefact from the spatial distribution proxy, which uses district level urban population to distribute on-road gasoline emissions. Gasoline vehicles mostly consist of two-, three- and four-wheeler private vehicles in use in urban areas. Therefore, in the present regional-scale inventory, it is based on population. However, improved road-based proxies for air-quality studies at urban scales are still necessary.

Overall, sources significantly influencing PM_2.5_ levels include residential biomass in all regions, open burning of agricultural residue in northern India and power plant and industrial coal combustion in eastern and southern India. In northern India, PM_2.5_ concentrations arise primarily from residential biomass combustion, followed by the open burning of agricultural residue. In contrast, in eastern and southern India, while residential biomass combustion is dominant, coal burning in the power and industrial sector is the next important source. Windblown dust contributes significantly to PM_2.5_ in north-western India, while anthropogenic dust (largely coal fly ash) contributes significantly to PM_2.5_ in eastern and southern India. Under an ambitious prospective policy scenario, promoting very large shifts away from traditional biomass technologies and coal-based electricity generation, significant reductions in PM_2.5_ levels are achievable in 2030 and 2050. Future air pollution is dominated by industrial process emissions, reflecting a larger expansion in industrial rather than residential energy demand. Potential future contributions of anthropogenic dust are large, while those from transportation and distributed diesel sources are also projected to increase substantially, although to a small extent in comparison to other sources.

## 5 Conclusions

This work represents the most comprehensive examination to date of a systematic analysis of source influence, including all sources, on present and future air pollution on a regional scale over India. Elevated annual mean PM_2.5_ concentrations are a pan-India problem, with a regional character, and are not limited to urban areas or megacities. Under present-day emissions, simulations indicate that population-weighted mean concentrations in most states are above the national PM_2.5_ standard. Under present-day (2015) emissions, residential biomass fuel use for cooking and heating is the largest single sector influencing outdoor air pollution across most of India. The dominance of residential biomass fuel emissions is an important underlying cause for the regional nature of air pollution in India because of the widely dispersed and distributed nature of this uncontrolled source. Agricultural residue burning is the next important source, especially in north-western and northern India. This large influence on an annual basis suggests even larger impacts during the burning periods (typically April–May and October–December). In eastern and peninsular India, the influence of coal burning in thermal power plants and industry follows that of residential biomass combustion. Anthropogenic dust (including coal fly ash, mineral matter from combustion and urban fugitive dust), brick production and vehicular emissions are also important sources. Overall, the findings suggest a large regional background of PM_2.5_ pollution (from residential biomass, agricultural residue burning and power plant and industrial coal) underlying that from local sources (transportation, brick kilns, distributed diesel) in peri-urban areas and megacities.

If no action is taken, population exposure to PM_2.5_ is likely to increase substantially in India by 2050. Evolution of emissions under current regulation (REF) and promulgated or proposed regulation (S2) yields a deterioration in future air quality in 2030 and 2050. Only under the S3 scenario of ambitious prospective regulation, not yet formulated, promoting a total shift away from traditional biomass technologies and a very large shift (80–85 %) to non-fossil electricity generation, is there an overall reduction in PM_2.5_ concentrations below 2015 levels, both in 2030 and 2050, with 20 states and 6 union territories projected to reach population-weighted mean concentrations below the national ambient air-quality standard. However, even under the most active reductions envisioned, the 2050 population-weighted mean exposure for the S3 scenario, excluding any impact from windblown mineral dust, is estimated to be nearly 3 times higher than the WHO Air Quality Guideline. Further exploration of air pollution mitigation measures must address the industrial sector, including process emissions, dispersed sources including waste burning and urban fugitive dust and traditional technologies in residential lighting and informal industry. This study shows that future emission increases in India, if realized, could have important implications for air pollution and climate change on regional and hemispheric scales. Importantly, a government-led initiative for detailed emission inventory development at national state and city levels is needed to support air-quality management. Incorporation of detailed Indian emissions, along with their rationalization to other Asian and global inventories, into multi-model studies over the Indian domain would provide insight into atmospheric processes that are still lacking in this region.

## Supplementary Material

SI

## Figures and Tables

**Table 1.** Description of source categories and sensitivity simulations.

	Sectors	Source categories	Acronym	Description of sensitivity simulations^a^	
1	Power plant coal	Thermal power plants	PCOL	Emissions from coal burning in power plants	
2	Industrial coal	Heavy and light industry	ICOL	Emissions from coal burning in heavy and light industries	
3	Total coal	Thermal power plants, heavy and light industry (sum of 1 and 2)	TCOL	Emissions from coal burning in electricity generation, heavy and light industry	
4	Transportation	Private (two-, three- and four- wheelers - gasoline), public (four-wheelers - diesel), freight (LDDVs^b^, HDDVs^c^) and railways	TRAN	Emissions from on-road and off-road transport including railways	
5	Distributed diesel	Agricultural pumps, tractors and DG^d^ sets	DSDL	Emissions from agricultural pumps, tractors and diesel generator sets	Sensitivity simulations
6	Residential biomass	Cooking, water heating and space heating	REBM	Emissions from residential biomass combustion for cooking and heating	
7	Brick production	Brick kilns	BRIC	Emissions from brick production	
8	Open burning	Agricultural residue burning	OBRN	Emissions from agricultural residue burning and forest fires	
9	Anthropogenic dust	Mineral matter from combustion and industry, urban fugitive dust	ADST	Emissions of anthropogenic dust	
10	Total dust	Windblown mineral dust and anthropogenic dust	TDST	Emissions of dust including windblown mineral dust and from anthropogenic activities	
11	Others	Residential lighting (kerosene), cooking (LPG^e^/kerosene), informal industry, waste burning and urban fugitive dust		No sensitivity run was carried out for source categories in this sector except for mineral matter from waste burning and urban fugitive dust (both accounted for in ADST)	No sensitivity simulation
12	Standard	Sum of sectors 1–11, except no. 3	STND	Standard emissions for the year 2015 from all sectors.	Standard simulation

a For each sensitivity simulation, emissions from individual sectors (nos. 1–10) are removed, respectively, from the standard emissions (no. 12). Sensitivity simulation results therefore depict the ambient PM_2.5_ concentrations with each emission sector shut off. The differences of the standard and sensitivity simulations were analysed to produce contributions of the individual sectors to ambient PM_2.5_ concentrations. The “others” sector was not separately addressed in sensitivity simulations. Meteorology was from the year 2012.

b LDDVs: light duty diesel vehicles.

c HDDVs: heavy duty diesel vehicles.

d DG: diesel generator.

e LPG: liquefied petroleum gas.

**Table 2.** Description of future scenarios.

Source sectors	REF: reference scenario	S2: aspirational scenario	S3: ambitious scenario
Thermal power	Low influx of renewable energy with large dominance of subcritical power plants.	Share of renewable energy (40 % by 2030) as targeted in India’s NDC with negligible flue gas desulfurization from a slow adoption of recent regulation (MoEFCC, 2015).	75–80 % of non-fossil-power generation (Anan- darajah and Gambhir, 2014; Shukla and Chaturvedi, 2012; Level 4, IESS, Niti Aayog, 2015); 80–95 % use of flue gas desulfurization.
Heavy and light industry	Set at present-day efficiency levels (58–75 %).	Modest increases in energy efficiency (62–84 %) under the Perform Achieve and Trade (PAT) scheme (Level 2, IESS, Niti Aayog, 2015).	Near complete shift to high efficiency (85–100%) industrial technologies (Level 4, IESS, Niti Aayog, 2015).
Transport	Present-day share of public and private vehicles.	Promulgated growth in public vehicle share (25–30%) (NTDPC, 2013; Guttikunda and Mohan, 2014; NITI Aayog, 2015), with slower shifts to BS-VI standards (MoRTH, 2016; ICRA, 2016).	Large shifts to public vehicles (40–60 %) (NITI Aayog, 2015), energy efficiency improvements in engine technology (MoP, 2015) and increased share of electric and CNG vehicle share (2050%) (NITI Aayog, 2015).
Brick and informal industry	Largely dominated by traditional technologies, such as Bull’s trench kilns and clamp kilns.	Modest increases in non-fired-brick walling materials (30–45 %) (UNDP, 2009; Sameer Maithel, personal communication, 2016).	Large share of non-fired-brick walling materials (40–70 %) and shift towards use of gasifiers in informal industries (65–80 %).
Residential	Minor shift (~ 40%) to energy-efficient technologies and fuels.	Slow shift (55 % in 2030 and 70 % in 2050) to energy-efficient technologies and fuels (Level 2, IESS, Niti Aayog, 2015).	Large shifts (90 % in 2030 and total in 2050) to LPG and electricity for cooking and heating devices (Level 4, IESS, Niti Aayog, 2015), with complete shift to electric and solar lamps for lighting (National Solar Mission, 2010).
Agricultural	No reduction in agricultural residue burning.	No reduction in agricultural residue burning.	Slow shift (35 % phase out by 2030) and complete phase-out (2050) of agricultural residue burning through a switch to mulching practices (Gupta, 2014).
