# Supplementary material for: Source influence on emission pathways and ambient PM2.5 pollution over India (2015–2050)
Source: Atmos Chem Phys. Author manuscript; Available in PMC 2021 Mar 5. (PMC7935015; doi:10.5194/acp-18-8017-2018)
Supplement: SI [file NIHMS1669561-supplement-SI.pdf]

Supplement of Atmos. Chem. Phys., 18, 8017–8039, 2018
https://doi.org/10.5194/acp-18-8017-2018-supplement
© Author(s) 2018. This work is distributed under
the Creative Commons Attribution 4.0 License.

Atmospheric Chemistry and Physics
Open Access

Supplement of

Source influence on emission pathways and ambient $PM_{2.5}$ pollution over India (2015–2050)

Chandra Venkataraman et al.

Correspondence to: Chandra Venkataraman (chandra@iitb.ac.in)

The copyright of individual parts of the supplement might differ from the CC BY 4.0 License.

List of Figures:

Figure S1. Methodology for estimation of future sectoral activity, apportionment to technology mix and related scenario based emissions........ 6
Figure S2. Sectoral Growth between 2015- 2050. Growth rates were computed based on analysis of existing data and reviewed literature........ 7
Figure S3. Energy Evolution in Scenarios REF, S2 and S3........ 13
Figure S4. Comparisons of national totals of SLCPs with HTAP_v2, REAS2.1 and ECLIPSE for 2008 and 2010........ 17
Figure S5. Percentage deviation in emissions of ECLIPSE V5a-CLE and GAINS-WEO2016-NPS from emissions of this study by sector........ 18
Figure S6. Top 20 polluted cities in India (2016)........ 21
Figure S7. Mean population-weighted ambient PM2.5 concentrations for 2015 and future scenarios. The bars represent the 95% Confidence Interval for the estimates........ 22

List of Tables:

Table S1. Spatial proxies used to distribute emissions........ 3
Table S2. Emissions for 2015 by state (MT/yr)........ 4
Table S3. Uncertainty Bounds (95% Confidence Levels) for Indian Emissions of Individual Pollutants by Sector........ 5
Table S4. Sectoral growth rates for 2015-2030 and 2030-2050........ 7
Table S5. Technology fraction for major emissions emitting sectors........ 9
Table S6. Specific energy per unit activity for each technology (PJ/activity)........ 11
Table S7. Energy demand for each technology (EJ/year)........ 13
Table S8. Emission factors of SLCP's, fine particulate matter and CO_2 (g/kg of fuel used)........ 15
Table S9. Representative Concentration Pathways (RCP) scenarios values over India........ 20
Table S10. Description of GEOS-CHEM NMVOC species........ 21

S1. Present day emissions at state-level and sectoral uncertainties in emissions

Table S1. Spatial proxies used to distribute emissions

<table>
<tr><th>Source Category/Activity</th><th>Proxies</th><th>Reference</th></tr>
<tr><td>Brick production</td><td>Distributed at district level using district-wise no. of household built using burnt bricks, excluding the cities with high population densities and distributing the emissions from those city grids in the surrounding grids.</td><td>Census, 2011</td></tr>
<tr><td>Food and agro processing (Jaggery making, cashewnut processing unit, tea and coffee drying, spices drying, silk reeling and dairy processing)</td><td>Jaggery making – district level sugarcane produced
Cashewnut, tea, coffee – distributed to specific districts with production as proxy
Spices drying – district level spices produced
Silk reeling – distributed to specific states (production) carrying this activity with rural population further at district level
Dairy processing – emissions distributed according to state production and further acc. to rural population at district level</td><td>Ministry of Agriculture;
Cashew Manufacturer’s Association;
Indian Tea Association;
Coffee Board of India;
Central silk board;
Department of animal husbandry</td></tr>
<tr><td>Thermal power, Cement, Fertilizer, Iron & Steel (ISP & Secondary producers), Non-ferrous, Refineries</td><td>Point sources with specific latitude and longitude known</td><td>Web source</td></tr>
<tr><td>Other industry, Iron & Steel (EAF, IF, Sponge iron)</td><td>Distributed at district level using urban population as proxy</td><td>Census, 2011</td></tr>
<tr><td>On-road gasoline vehicles & LDV diesel vehicles</td><td>Distributed at district level using urban population as proxy</td><td>Census, 2011</td></tr>
<tr><td>On-road diesel vehicles including HDV, Buses and Superemitters</td><td>Distributed on road network with following assumption: 40% National highway; 30% Golden Quadrilateral network; 20% State highway and 10% District roads and city grids</td><td>25 × 25km^2 Gridded shape-file showing various road networks</td></tr>
<tr><td>Residential cooking – solid fuel (fuelwood/ dungcake/crop residue/ coal)</td><td>Distributed at district level using no. of households using fuelwood, dungcake, crop residue and coal as fuel</td><td rowspan="3">Census, 2011</td></tr>
<tr><td>Residential cooking – LPG/ kerosene/biogas</td><td>Distributed at district level using no. of households using LPG, kerosene and biogas as fuel</td></tr>
<tr><td>Residential lighting – kerosene</td><td>Distributed at district level using rural population</td></tr>
<tr><td>Agricultural residue burning</td><td>Distributed at district level using district-wise cereals and sugarcane produced during 2010-11</td><td>Ministry of Agriculture, 2011</td></tr>
<tr><td>Agriculture diesel use</td><td>Distributed at district level using district-wise area cultivated during 2010-11</td><td>Ministry of Agriculture, 2011</td></tr>
</table>

Table S2. Emissions for 2015 by state (MT/yr)

States	PM2.5	BC	OC	SO2	NOX	NMVOC	NH3*
Andaman and Nicobar	0.124	0.001	0.003	0.426	0.460	0.032	0.001
Arunachal Pradesh	0.006	0.001	0.002	0.000	0.002	0.010	0.018
Assam	0.144	0.031	0.048	0.067	0.096	0.266	0.235
Bihar	0.747	0.117	0.245	0.331	0.338	1.754	0.548
Chandigarh	0.067	0.012	0.029	0.008	0.011	0.122	0.000
Chhattisgarh	0.327	0.020	0.024	0.643	0.704	0.124	0.239
Dadra & Nagar Haveli	0.002	0.000	0.000	0.003	0.003	0.006	0.002
Diu and Daman	0.001	0.000	0.000	0.000	0.002	0.002	0.002
Goa	0.008	0.002	0.001	0.009	0.011	0.013	0.004
Gujarat	0.505	0.073	0.089	0.904	0.701	0.881	0.529
Haryana	0.305	0.041	0.084	0.216	0.322	0.530	0.295
Himachal Pradesh	0.030	0.007	0.009	0.004	0.023	0.048	0.063
Jammu and Kashmir	0.058	0.011	0.019	0.007	0.026	0.101	0.085
Jharkhand	0.230	0.043	0.069	0.186	0.181	0.404	0.346
Karnataka	0.439	0.065	0.109	0.338	0.432	0.702	0.551
Kerala	0.142	0.029	0.034	0.107	0.133	0.271	0.151
Lakshadweep	0.002	0.001	0.001	0.000	0.004	0.005	0.000
Madhya pradesh	0.498	0.074	0.107	0.532	0.678	0.706	0.795
Maharashtra	0.437	0.070	0.073	0.570	0.709	0.917	0.823
Manipur	0.013	0.003	0.005	0.001	0.022	0.023	0.027
Meghalaya	0.009	0.002	0.002	0.002	0.008	0.010	0.036
Mizoram	0.195	0.029	0.082	0.011	0.022	0.372	0.008
Nagaland	0.017	0.003	0.004	0.002	0.014	0.027	0.020
NCT Delhi	0.060	0.011	0.009	0.057	0.097	0.132	0.011
Orissa	0.372	0.050	0.077	0.489	0.319	0.472	0.354
Puducherry	0.004	0.001	0.001	0.003	0.006	0.008	0.002
Punjab	0.450	0.050	0.133	0.189	0.405	0.768	0.491
Rajasthan	0.515	0.078	0.131	0.680	0.536	0.681	0.618
Seemandhra	0.323	0.051	0.082	0.220	0.265	0.572	0.567
Sikkim	0.003	0.001	0.001	0.000	0.001	0.004	0.006
Tamilnadu	0.441	0.065	0.089	0.461	0.576	0.685	0.460
Telangana	0.232	0.036	0.059	0.158	0.190	0.410	0.332
Tripura	0.019	0.003	0.007	0.001	0.008	0.033	0.032
Uttar pradesh	1.676	0.231	0.508	0.915	1.510	3.579	1.428
Uttarakhand	0.043	0.010	0.014	0.006	0.048	0.090	0.098
West Bengal	0.656	0.098	0.186	0.544	0.637	1.078	0.599
**India**	**9.101**	**1.320**	**2.337**	**8.091**	**9.498**	**15.839**	**9.777**

**NH3 emissions at state-level are obtained from the gridded files from MIX Asian Inventory (http://www.meicmodel.org/dataset-mix.html) (Li et al., 2017)*

Table S3. Uncertainty Bounds (95% Confidence Levels) for Indian Emissions of Individual Pollutants by Sector

Sector	NO_X	SO_2	$PM_{2.5}$	NMVOC
Industry	[-85%, +256%]	[-22%, +26%]	[-81%, +217%]	[-80%, +209%]
Transport	[-63%, +122%]	[-71%, +157%]	[-54%, +91%]	[-59%, +107%]
Residential	--	[-59%, +107%]	[-61%, +113%]	[-66%, +133%]
Agricultural	[-60%, +111%]	[-58%, +105%]	[-46%, +70%]	[-63%, +121%]
Informal industry	[-85%, +260%]	[-10%, +11%]	[-74%, +173%]	[-79%, +204%]
**Total Emissions**	**[-65%, +125%]**	**[-20%, +24%]**	**[-49%, +78%]**	**[-44%, +66%]**

Uncertainties in the activity rates were calculated analytically, assuming normal distribution for the underlying uncertainties in all input quantities. For each input: (a) the mean and standard deviation calculated from a set of available (three or more) data points; (b) upper and lower bounds assumed based on two data points; or (c) a representative uncertainty assumed from similar data, where only one data-point exists. Uncertainty in the emission factors was estimated from the standard deviation in the set of compiled emission factors of a particular pollutant from a particular fuel technology combination. If the emission factor being used was taken from a single reported source, the reported rating was quantified using the percentage errors cited in IPCC (2006a,b) and EMEP (2009). The measured emission factors with unspecified uncertainties were assigned the highest-known uncertainty for the same pollutant and those from similar technologies. Wherever emission factor measurements for a technology were not available an emission factor from a similar technology was chosen and assigned 100% uncertainty (<5% of the technologies fall under this category, including fluidized bed combustors and sponge-iron kilns). A spreadsheet-based approach was developed for combining uncertainties in activity rates and emission factors. A normal/lognormal distribution was assumed for when standard deviation was less/greater than 30% of the mean. Uncertainty propagation in the product of two variables was followed using the sum-of-quadrature rule, calculated analytically. The upper and lower emission bounds were calculated using the resultant lognormal parameters (geometric mean and geometric standard deviation).

S2. Future emission pathways

S2.1. Methodology

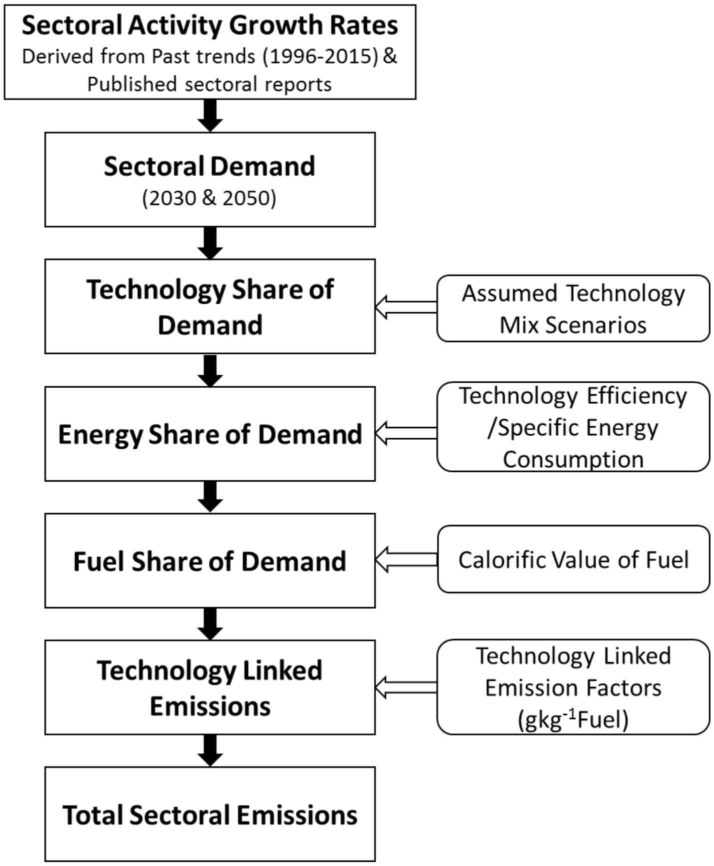

Figure S1. Methodology for estimation of future sectoral activity, apportionment to technology mix and related scenario based emissions.

S2.2. Evolution of sectoral demand

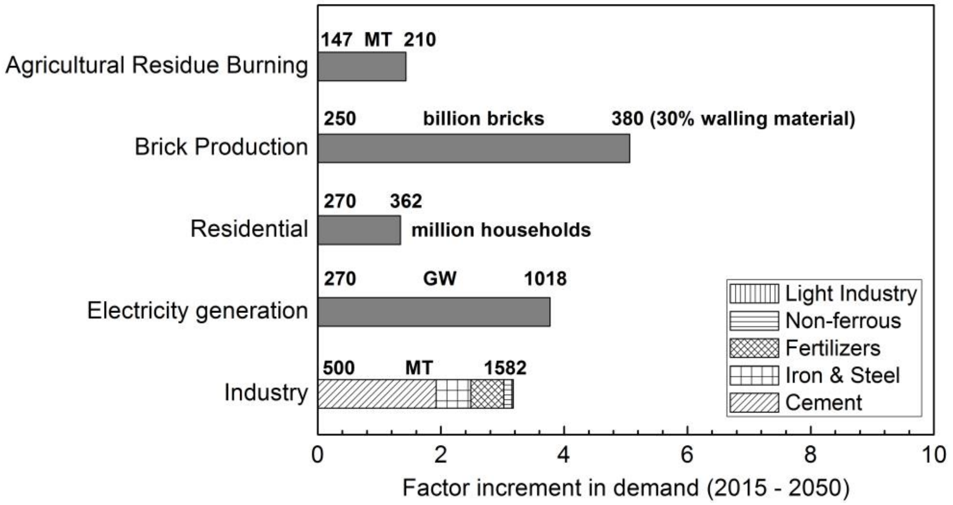

Figure S2. Sectoral Growth between 2015- 2050. Growth rates were computed based on analysis of existing data and reviewed literature.

Table S4. Sectoral growth rates for 2015-2030 and 2030-2050

**Sectors**	**Activity Name**	**Activity**	**Growth rates in % per year**						
		**2015**		**2015 - 2030**			**2030 - 2050**		
			**Growth rate from 2000-2015 data**	**IESS[a]Growth Rate**	**Published growth rate**	**This Study**	**IESS[a]Growth Rate**	**Published growth rate**	**This Study**
Electricity generation	Installed capacity (GW)	270	6.89	6.31	6.7[b]	**6.63**	1.84	NIL	**1.84**
Industry	Production (MT)								
	Cement	215	5.06	5.63	7.08[c]	**6.07**	2.86	NIL	**3.1**
	Iron and steel	88	4.49	8.03	3.26[d]	**4.5**	2.93	NIL	**2.5**
	Fertilizer	190	1.77	1.04	2.86[e]	**2.32**	0.02	NIL	**0.04**
	Non-ferrous	4	6.65	9.74	11.3[f]	**11.3**	6.77	NIL	**6.23**
Brick production	Number of bricks (in billion) (similar to construction growth)	250	NIL	NIL	6.6[g]	**6.6**	NIL	NIL	**3.37**
Transport	Passenger-kilometre (in billion)	9997	6.54*	5.02	NIL	**5.78**	2.42	NIL	**2.89**
	Freight-kilometre (in billion)	2564	3.61	-	NIL	**3.61**	-	NIL	**1.8**
Residential	Household number (in million) (similar to population growth)	270	1.39	1.88	1.1[h]	**1.25**	1.57	0.47[h]	**0.53**
Agriculture	Crop production (KT)	578	1.02	NIL	~ 1 to 1.1[i]	**1.02**	NIL	NIL	**1.02**

	(i.e. cereal produced)								

* Growth rate calculated for data from 1996 - 2015

Sources: [Short forms in brackets]

a) India Energy Security Scenarios 2047, NITI Aayog, Govt. of India, 2015 [NITI Aayog 2015]

b) Data from the Ministry of Environment, Forest and Climate Change (MoEFCC) analysed by the Prayas Energy Group, 2011 [MoEFCC, 2011]

c) International Energy Agency, 2009 [IEA, 2009]

d) Dr.A.S.Firoz, Economic Research Unit, 2014 [Firoz, 2014]

e) Industry Group for Petroleum & Natural Gas Regulatory Board, 2013 [PNGRB, 2013]

f) Prof. K.S.S. Murthy, Gen. Secretary, Aluminium Association of India, 2014 [Murthy, 2014]

g) Maithelet. al., Study report prepared by Green Knowledge Solutions, New Delhi, 2012 [Maithel et al., 2012]

h) Shukla, P. R., et al. "Low Carbon Society Vision 2050. India. Indian Institute of Management Ahmedabad." National Institute for Environmental Studies, Kyoto University and Mizuho Information & Research Institute,2009 [Shukla et al., 2009]

i) Ray, D.K., Mueller, N.D., West, P.C. and Foley, J.A., 2013. Yield trends are insufficient to double global crop production by 2050. PloS one, 8(6), p.e66428. [Ray et. al., 2013]

S2.3 Evolution of technology mix

In 2015, power generation was almost entirely from subcritical pressure thermal power plants with an average gross efficiency of 30.5% (IEP, 2006; IESS, NITI Aayog 2015). A switch to more efficient technologies such as supercritical (SC), ultra-supercritical (USC), and integrated gasification combined cycle (IGCC) is expected in future. For 2030 and 2050, respectively, the non-fossil shares were assumed to be 30% and 40% in REF, 40% and 60% in S2, and 75% and 80% in S3. The assumed technology mix in S2 follows the NDC’s proposed non-fossil share of 2030. In S3, it is consistent with high efficiency-low carbon growth cases in earlier studies (Anandarajah and Gambhir 2014; Shukla and Chaturvedi 2012; Level 4, IESS, Niti Aayog, 2015). The transition of thermal power plants sub-critical boiler technology to more efficient technologies like super-critical, ultra-super-critical and integrated gasification combined cycle (IGCC) is based on published scenarios (IEP, 2006; IESS, Niti Aayog, 2015).

Emissions from on-road vehicles are based from a previous study (Pandey and Venkataraman, 2014). The detailed list of vehicle category is included in the study (Table 3, Pandey and Venkataraman, 2014). Two-wheelers contribute the most to the fleet of private vehicles with approximately 82% share, followed by passenger cars (15%) and three-wheelers (3%). For present day, all vehicles are assumed to be compliant with BS III standards with 2 wheelers having the highest emission levels for PM2.5 followed by three wheelers (0.5 times lower) and gasoline cars (0.1 times lower). Future shifts to BS IV and BS VI emission standards lead to reductions in emission levels by 80% and 90% respectively. The emissions standards for vehicular emissions in India are based on European standards. The values of emission limits as prescribed for BS IV and BS VI are equivalent to those in EURO IV and EURO VI, while the US standards are more stringent than BS standards (DieselNet, 2018). In the transport sector, current technology shares are 81% private vehicles (two-wheeler, three-wheeler and cars) and 19% public vehicles (buses and taxis) (Pandey and Venkataraman, 2014). The share of private vehicles is projected to increase in a reference scenario till 2030, especially for two-wheelers and cars (NTDPC, 2013; Guttikunda and Mohan, 2014). However, beyond 2030, as GDP stabilizes, no further increase in private vehicle share is assumed, with a greater demand for public transport. Therefore, in the S2 scenario, private vehicle share is assumed as 75% and 70% in 2030 and 2050, respectively. For S3 private vehicle share is assumed to decrease rapidly to 60% in 2030 and 40% in 2050 in consistent with Level 2 of IESS (NITI Aayog, 2015) (Table S5). For future emissions, Auto Fuel Policy (Auto Fuel Policy Vision 2025, 2014) recommendations were applied, wherein 2/3-wheelers were proposed to have Bharat Stage (BS)-IV standards from 1st April 2015, light and heavy duty diesel vehicles to have BS-Va and BS-Vb. There is a recent proposal to leapfrog directly to BS-VI for all on-road vehicle categories by 2020 (MoRTH, 2016). However, scenarios used here, do not reflect such a quick change, keeping the share of BS-VI at modest levels owing to expected delays in availability of BS-VI compliant fuels and/or difficulties in making the technologies adaptive to Indian road conditions as well as cost-effective (ICRA, 2016), along with the use of non-BS-VI compliant vehicles in peri-urban and rural areas. In the transport sector, engine efficiency improvements are not foreseen to have significant increases across technologies (e.g. across BS-III to BS-VI) as these standards primarily govern the control

of emissions of air pollutants. Until 2015 there were no fuel economy standards for India. However, energy efficiency improvement are assumed over the years in the S3 scenario keeping in mind the recently proposed fuel economy targets (MoP, 2015)

In the brick sector, currently 76% of total bricks are produced by Bull's trench kilns (BTK) and 21% by clamp kilns. Clamp kilns are highly polluting, with sun-dried bricks, stacked alternately with layers of powdered fuel, allowed to smolder until the bricks are baked. The demand for non-fired-brick walling materials is currently negligible, but expected to rise (10-25% in REF, 30-45% in S2 and 40-75% in S3 for 2030-2050), from increased availability of hollow-block technology and the governmental incentives for fly-ash bricks (UNDP, 2009). For fired bricks, cleaner technologies include a retrofit to existing Bull's trench kilns, called zig-zag firing, or significantly more capital intensive, vertical shaft brick kilns (VSBK) which have increased efficiency. For small clamp kilns, it is believed that regulation may not be effective, so a constant activity level, but a decreasing share was assumed in future, with new cleaner technologies filling growing demand (personal communication, Maithel, 2015).

Evolution of technologies in informal industry from say traditional wood furnaces, presently supplying all energy requirements, to gasifier and LPG based technologies is assumed to increase in 2030 and 2050 respectively, to 20% and 35% in S2 and 65% and 80% in S3 (Table S5).

India's rural population largely depends on biomass fuels for cooking and lighting (Venkataraman et al. 2010). Although India has introduced improved biomass cook-stoves to improve fuel efficiency and to reduce smoke exposure using chimneys or combustion improvements, further technological improvements or alternatives are required to reach LPG-like emission levels to reduce disease risk due to household biomass burning. The REF scenario assumes an increasing penetration rate of liquefied petroleum gas (LPG) and piped natural gas (PNG) typical of 1995–2015 (Pandey et al. 2014). In the S2 and S3 scenarios, assumed future switch in residential energy to use of LPG/PNG or low-emission biomass gasifier stoves and biogas, is consistent with energy efficiency increases proposed in Levels 2 and 4 of the IESS (NITI Aayog 2015). We use lower rates of clean technology adoption in the residential sector in both the REF and S2 scenarios, because no current legislation or standards target this sector, but a complete switch away from traditional biomass fuels in S3. In case of lighting, 37% usage is of highly polluting kerosene wick lamps and lanterns, which emit large amounts of black carbon (Lam et al. 2012), while the balance is of electricity, with less than 1% solar lamps. Residential lighting is assumed to shift from a modest present-day dependence on kerosene to a complete switch to electricity and solar lamps in 2030 and 2050 (National Solar Mission 2010), a change expected with a national promotion of renewable energy.

In the agricultural sector it is assumed, based on satellite active fire cycles in agricultural land-use areas(Venkataraman et al., 2006), that residues of cereal and sugarcane are burned in field. Gupta (2014) indicated greater mechanization of agriculture, with decrease in amounts of residue, but increase in incidence of field burning, needed to clear the rubble consisting of 6-12 inch stalks, before sowing. Mulching technology was reported to allow sowing even through rubble and loosely spread residue, thus avoiding burning for field clearing. The present work applies different levels of mulching, replacing field burning, in future years (Table S5).

Table S5. Technology fraction for major emissions emitting sectors

Sector	Source Categories	TechMix		REF		S2		S3	
			2015	2030	2050	2030	2050	2030	2050
Thermal power	Thermal power	Fossil-fuel energy	0.70	0.70	0.60	0.60	0.40	0.25	0.2
		Coal fraction	0.61	0.59	0.48	0.48	0.24	0.20	0.12
		Gas fraction	0.09	0.11	0.12	0.12	0.16	0.05	0.08
		Non-carbon energy	0.30	0.30	0.40	0.40	0.60	0.75	0.80

		Sub-critical	1.00	0.90	0.70	0.65	0.55	0.40	0.30
		Super-critical	0.00	0.10	0.15	0.15	0.20	0.15	0.15
		Ultra super critical	0.00	0.00	0.10	0.12	0.15	0.20	0.20
		IGCC	0.00	0.00	0.05	0.08	0.10	0.25	0.35
Heavy Industry	Cement	PAT	0.72	0.72	0.72	0.77	0.83	0.90	1.00
		Non-PAT	0.28	0.28	0.28	0.23	0.17	0.10	0.00
	Iron and steel	PAT	0.56	0.58	0.60	0.62	0.69	0.85	1.00
		Non-PAT	0.44	0.42	0.40	0.38	0.31	0.15	0.00
	Fertilizer	PAT	0.75	0.75	0.75	0.79	0.84	0.95	1.00
		Non-PAT	0.25	0.25	0.25	0.21	0.16	0.05	0.00
	Non-ferrous	PAT	0.69	0.69	0.70	0.76	0.83	0.90	1.00
		Non-PAT	0.31	0.31	0.30	0.24	0.17	0.10	0.00
Light Industry		PAT	0.30	0.35	0.40	0.50	0.70	0.85	1.00
		Non-PAT	0.70	0.65	0.60	0.50	0.30	0.15	0.00
Brick and informal industry	Brick Production	BTK	0.76	0.50	0.35	0.40	0.20	0.20	0.00
		Clamps	0.21	0.20	0.05	0.15	0.05	0.05	0.00
		Zig-zag firing	0.02	0.15	0.15	0.10	0.10	0.20	0.10
		Hollow	0.01	0.05	0.20	0.05	0.20	0.15	0.20
		Non-fired bricks	0.00	0.10	0.25	0.30	0.45	0.40	0.70
	Informal industry	Trad. Biofuel	1.00	0.90	0.75	0.80	0.65	0.35	0.20
		Gasifier	0.00	0.10	0.25	0.20	0.35	0.65	0.80
Transport	Passenger - Private	Private Vehicles	0.81	0.81	0.81	0.75	0.70	0.60	0.40
		Gasoline	0.97	0.94	0.87	0.88	0.70	0.62	0.30
		BS III	1.00	0.39	0.00	0.08	0.00	0.00	0.00
		BS IV	0.00	0.39	0.00	0.30	0.00	0.30	0.00
		BS V	0.00	0.22	0.71	0.47	0.41	0.30	0.25
		BS VI	0.00	0.00	0.29	0.15	0.59	0.40	0.75
		CNG	0.03	0.05	0.10	0.08	0.20	0.13	0.20
		Electric	0.00	0.01	0.03	0.04	0.10	0.25	0.50
	Passenger - Public	Public Vehicles	0.19	0.19	0.19	0.25	0.30	0.40	0.60
		Diesel	0.98	0.90	0.90	0.82	0.70	0.60	0.40
		BS III	1.00	0.58	0.00	0.15	0.00	0.00	0.00
		BS IV	0.00	0.24	0.00	0.25	0.00	0.12	0.00
		BS V	0.00	0.18	0.59	0.35	0.29	0.38	0.21
		BS VI	0.00	0.00	0.41	0.25	0.71	0.50	0.79
		CNG	0.02	0.05	0.05	0.10	0.20	0.20	0.30
		Electric	0.00	0.05	0.05	0.08	0.10	0.20	0.30
	Freight	Diesel (BS-III)	0.58	0.30	0.00	0.25	0.00	0.20	0.00
		(BS-IV)	0.00	0.23	0.12	0.20	0.02	0.15	0.00
		(BS-V)	0.00	0.05	0.35	0.10	0.40	0.15	0.15
		(BS-VI)	0.00	0.00	0.08	0.00	0.08	0.00	0.30
Residential	Cooking	Trad. Biofuel	0.68	0.61	0.55	0.45	0.30	0.10	0.01
		Gasifier	0.00	0.03	0.05	0.13	0.20	0.35	0.20
		Kerosene	0.03	0.00	0.00	0.00	0.00	0.00	0.00
		LPG	0.29	0.35	0.38	0.37	0.42	0.45	0.61
		Electricity	0.00	0.01	0.02	0.05	0.08	0.10	0.18
	Lighting	Kerosene	0.42	0.34	0.26	0.10	0.05	0.00	0.00
		Electricity and solar	0.58	0.66	0.74	0.90	0.95	1.00	1.00
	Space heating	Wood	1.00	0.95	0.85	0.90	0.80	0.85	0.70
		Electric & solar	0.00	0.05	0.15	0.10	0.20	0.15	0.30
	diesel genset	Diesel	1.00	0.95	0.85	0.90	0.80	0.85	0.75
		Electric & solar	0.00	0.05	0.15	0.10	0.20	0.15	0.25
Agriculture	Agr.res.burn	Open Residue Burning	1.00	1.00	1.00	1.00	1.00	0.65	0.00

		Deep sowing mulching tech	0.00	0.00	0.00	0.00	0.00	0.35	1.00
	Agr. Pumps	Diesel	0.32	0.27	0.20	0.10	0.05	0.05	0.00
		Electric & solar	0.68	0.73	0.80	0.90	0.95	0.95	1.00
	Agr. Tractors	Diesel	1.00	1.00	1.00	1.00	1.00	1.00	1.00

S2.4. Evolution of specific energy and total energy consumption

Different technologies are matched with corresponding specific energy per unit activity (Table S6), related to each technology type. In technology evolution, a given technology may improve in efficiency with time or may be replaced with higher efficiency- lower emissions technology at greater rates with time. Both these possibilities are captured in the assumptions, with no efficiency improvement with time characterizing REF, but with increasing efficiency improvements with time (in 2030 and 2050) characterizing S2 and S3 scenarios (Table S6). Thus in scenarios with high-efficiency energy technologies, there is a reduction of total energy consumption despite increase in activity.

In thermal power sector, the shift in energy efficiency is seen across the technologies from sub-critical plants being the least efficient to plants using integrated gasified combined cycle having the highest efficiency. Under REF scenario, the individual technologies are not assumed to undergo any improvement in their energy utilization. For S2 and S3, each technology is assumed to have better energy efficiency by 10% in 2030 and 15% in 2050. This evolution of energy efficiency in power plants is governed by the Perform, Achieve and Trade (PAT) scheme. To nurture energy efficiency in industries, Bureau of Energy Efficiency (BEE) under Ministry of Power launched the 'Perform, Achieve and Trade' (PAT) scheme under the National Mission on Enhanced Energy Efficiency (NMEEE) since July, 2012 (MoP, 2012; IESS, NITI Aayog, 2015). Under this scheme, every industry (includes power plants and heavy industries, referred to as "designated consumers" in the scheme) must meet a certain energy efficiency target by implementing appropriate and timely technological reforms. Thus, for industries also, the specific energy per unit activity is representative of the level of penetration of the PAT scheme across different industries over time under each scenario.

Table S6. Specific energy per unit activity for each technology (PJ/activity)

Sector	Source Categories	TechMix	Acitivity		REF		S2		S3	
			(units)	2015	2030	2050	2030	2050	2030	2050
Thermal power	Thermal power	Sub-critical-coal	GW	68.24	68.24	68.24	61.41	58.00	61.41	58.00
		Super-critical-coal	GW	60.79	60.79	60.79	54.71	51.67	54.71	51.67
		Ultra super critical-coal	GW	54.05	54.05	54.05	48.65	45.94	48.65	45.94
		IGCC-coal	GW	52.01	52.01	52.01	46.81	44.21	46.81	44.21
		Sub-critical-gas	GW	39.00	39.00	39.00	35.10	33.15	35.10	33.15
		Super-critical-gas	GW	34.75	34.75	34.75	31.27	29.53	31.27	29.53
		Ultra super critical-gas	GW	30.89	30.89	30.89	27.81	26.26	27.81	26.26
		IGCC-gas	GW	29.73	29.73	29.73	26.75	25.27	26.75	25.27
Heavy Industry	Cement	PAT	Million Ton	4.47	4.47	4.47	4.02	3.80	4.02	3.80
		Non-PAT	Million Ton	4.56	4.56	4.56	4.10	3.88	4.10	3.88
	Iron and steel	PAT	Million Ton	25.62	25.62	25.62	23.06	21.78	23.06	21.78
		Non-PAT	Million Ton	34.83	34.83	34.83	31.35	29.61	31.35	29.61

	Fertilizer	PAT	Million Ton	1.30	1.30	1.30	1.17	1.10	1.17	1.10
		Non-PAT	Million Ton	1.39	1.39	1.39	1.25	1.18	1.25	1.18
	Non-ferrous	PAT	Million Ton	189.27	189.27	189.27	170.35	160.88	170.35	160.88
		Non-PAT	Million Ton	280.24	280.24	280.24	252.21	238.20	252.21	238.20
Light Industry[1]		PAT		n.a.	n.a.	n.a.	n.a.	n.a.	n.a.	n.a.
		Non-PAT		n.a.	n.a.	n.a.	n.a.	n.a.	n.a.	n.a.
Brick and informal industry	Brick Production	BTK	Billion Bricks	3.75	3.75	3.75	3.00	2.81	3.00	2.81
		Clamps	Billion Bricks	7.91	7.91	7.91	6.33	5.93	6.33	5.93
		Zig-zag firing	Billion Bricks	2.25	2.25	2.25	1.80	1.68	1.80	1.68
		Hollow	Billion Bricks	1.67	1.67	1.67	1.34	1.25	1.34	1.25
		Non-fired bricks	Billion Bricks	0.00	0.00	0.00	0.00	0.00	0.00	0.00
	Informal industry	Trad. Biofuel	Million Ton	14.65	14.65	14.65	11.72	10.98	11.72	10.98
		Gasifier	Million Ton	8.79	8.79	8.79	7.03	6.59	7.03	6.59
	Passenger - Private	Gasoline -BS III	Billion Pass. Km	0.08	0.08	0.08	0.08	0.08	0.07	0.06
		BS IV	Billion Pass. Km	0.08	0.08	0.08	0.08	0.08	0.07	0.06
		BS V	Billion Pass. Km	0.08	0.08	0.08	0.08	0.08	0.07	0.06
		BS VI	Billion Pass. Km	0.08	0.08	0.08	0.08	0.08	0.07	0.06
		CNG	Billion Pass. Km	0.14	0.14	0.14	0.14	0.14	0.13	0.11
		Electric	Billion Pass. Km	0.00	0.00	0.00	0.00	0.00	0.00	0.00
	Passenger - Public	Diesel-BS III	Billion Pass. Km	0.51	0.51	0.51	0.51	0.51	0.46	0.38
		BS IV	Billion Pass. Km	0.51	0.51	0.51	0.51	0.51	0.46	0.38
		BS V	Billion Pass. Km	0.51	0.51	0.51	0.51	0.51	0.46	0.38
		BS VI	Billion Pass. Km	0.51	0.51	0.51	0.51	0.51	0.46	0.38
		CNG	Billion Pass. Km	0.62	0.62	0.62	0.62	0.62	0.56	0.47
		Electric	Billion Pass. Km	0.00	0.00	0.00	0.00	0.00	0.00	0.00
	Freight	Diesel (BS-III)	Billion Ton Km	1.19	1.19	1.19	1.19	1.19	1.07	0.89
		(BS-IV)	Billion Ton Km	1.19	1.19	1.19	1.19	1.19	1.07	0.89
		(BS-V)	Billion Ton Km	1.19	1.19	1.19	1.19	1.19	1.07	0.89
		(BS-VI)	Billion Ton Km	1.19	1.19	1.19	1.19	1.19	1.07	0.89
Residential	Cooking	Trad. Biofuel	Million HH	36.28	36.28	36.28	36.28	36.28	7.26	5.44
		Gasifier	Million HH	21.77	21.77	21.77	21.77	21.77	4.35	3.27
		Kerosene	Million HH	15.78	15.78	15.78	15.78	15.78	14.20	12.62
		LPG	Million HH	8.04	8.04	8.04	8.04	8.04	7.23	6.43
		Electricity	Million HH	0.00	0.00	0.00	0.00	0.00	0.00	0.00
	Lighting	Kerosene	Million HH	0.92	0.92	0.92	0.92	0.92	0.83	0.74
		Electricity and solar	Million HH	0.00	0.00	0.00	0.00	0.00	0.00	0.00
	Space heating	Wood	Million Ton	14.90	14.90	14.90	14.90	14.90	2.98	2.24
		Electric & solar	Million Ton	0.00	0.00	0.00	0.00	0.00	0.00	0.00
	diesel genset	Diesel	kTon	0.04	0.04	0.04	0.04	0.04	0.04	0.03
		Electric & solar	kTon	0.00	0.00	0.00	0.00	0.00	0.00	0.00

Agriculture	Agr.res.burn	Open Residue Burning	Million Ton	14.90	14.90	14.90	14.90	14.90	14.15	13.41
		Deep sowing mulching tech	Million Ton	0.00	0.00	0.00	0.00	0.00	0.00	0.00
	Agr. Pumps	Diesel	Million no.	67.57	67.57	67.57	67.57	67.57	60.81	50.68
		Electric & solar	Million no.	0.00	0.00	0.00	0.00	0.00	0.00	0.00
	Agr. Tractors	Diesel	Million no.	33.31	33.31	33.31	33.31	33.31	29.98	24.99

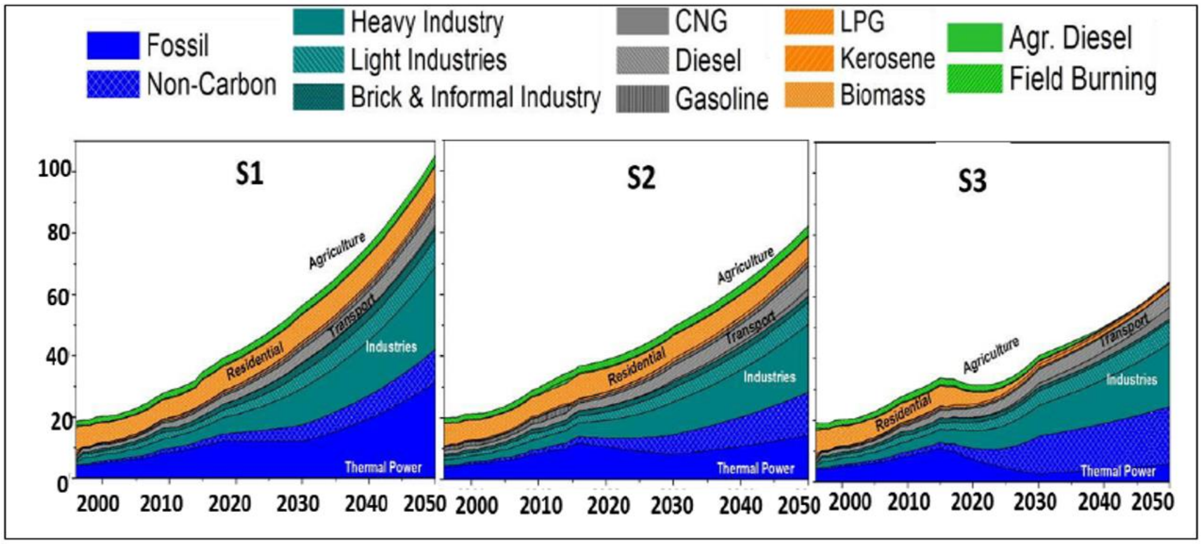

Figure S3. Energy Evolution in Scenarios REF, S2 and S3

Much of the energy demand in S1 is from electricity generation which is majorly fossil fueled, industry (coal and biomass fueled), in residential biomass is dominantly used as fuel. In scenarios S2 and S3 use of energy efficient technologies like Non-carbon fuel use thermal power, PAT implementation in industries and cleaner technologies in brick production, LPG use in residential and energy efficient standards in transport can help to lower the energy demand.

Table S7. Energy demand for each technology (EJ/year)

			Energy demand (EJ)					
Sector	Source Categories	TechMix	REF		S2		S3	
			2030	2050	2030	2050	2030	2050
Electricity generation	Electricity generation	Non-carbon energy	5.35	10.92	6.42	13.92	12.04	18.56
		Sub-critical-coal	8.76	23.35	4.65	7.80	1.19	2.13
		Super-critical-coal	0.37	4.46	0.41	1.09	0.17	0.41
		Ultra super critical-coal	0.00	2.64	0.37	0.92	0.26	0.61
		IGCC-coal	0.00	1.27	0.26	0.64	0.34	1.12
		Sub-critical-gas	2.78	3.34	1.94	2.97	0.50	0.81
		Super-critical-gas	0.12	0.64	0.17	0.42	0.07	0.16
		Ultra super critical-gas	0.00	0.38	0.15	0.35	0.11	0.23

		IGCC-gas	0.00	0.18	0.11	0.24	0.14	0.43
Heavy Industry	Cement	PAT	1.67	3.08	1.61	3.02	1.88	3.64
		Non-PAT	0.66	1.22	0.49	0.63	0.21	0.00
	Iron and steel	PAT	2.54	4.31	2.45	4.21	3.35	6.10
		Non-PAT	2.50	3.91	2.04	2.57	0.80	0.00
	Fertilizer	PAT	0.26	0.26	0.25	0.25	0.30	0.30
		Non-PAT	0.09	0.09	0.07	0.05	0.02	0.00
	Non-ferrous	PAT	2.54	8.62	2.51	8.69	2.98	10.47
		Non-PAT	1.69	5.47	1.18	2.63	0.49	0.00
Light Industry		PAT	1.81	3.70	2.58	6.48	3.95	7.08
		Non-PAT	3.35	5.56	2.58	2.78	0.70	0.00
Brick and informal industry	Brick Production	BTK	1.22	1.66	0.78	0.71	0.39	0.00
		Clamps	1.03	0.50	0.62	0.38	0.21	0.00
		Zig-zag firing	0.22	0.43	0.12	0.21	0.23	0.21
		Hollow	0.05	0.42	0.04	0.32	0.13	0.32
		Non-fired bricks	0.00	0.00	0.00	0.00	0.00	0.00
	Informal industry	Trad. Biofuel	0.44	0.45	0.31	0.29	0.14	0.09
		Gasifier	0.03	0.09	0.05	0.09	0.15	0.21
	Passenger - Private	Gasoline -BS III	0.56	0.00	0.09	0.00	0.00	0.00
		BS IV	0.56	0.00	0.37	0.00	0.18	0.00
		BS V	0.30	1.64	0.58	0.66	0.18	0.07
		BS VI	0.00	0.66	0.19	0.95	0.25	0.22
		CNG	0.13	0.51	0.20	0.82	0.24	0.35
		Electric	0.00	0.00	0.00	0.00	0.00	0.00
	Passenger - Public	Diesel-BS III	1.19	0.00	0.36	0.00	0.00	0.00
		BS IV	0.48	0.00	0.59	0.00	0.32	0.00
		BS V	0.36	2.10	0.83	1.26	0.96	0.79
		BS VI	0.00	1.47	0.59	3.15	1.28	2.99
		CNG	0.14	0.24	0.36	1.54	1.04	3.46
		Electric	0.00	0.00	0.00	0.00	0.00	0.00
	Freight	Diesel (BS-III)	1.26	0.00	1.05	0.00	0.76	0.00
		(BS-IV)	0.97	0.72	0.84	0.12	0.57	0.00
		(BS-V)	0.21	2.10	0.42	2.41	0.57	0.68
		(BS-VI)	0.00	0.48	0.00	0.48	0.00	1.35
Residential	Cooking	Trad. Biofuel	7.21	7.22	5.32	3.94	0.24	0.02
		Gasifier	0.21	0.39	0.92	1.58	0.50	0.24
		Kerosene	0.00	0.00	0.00	0.00	0.00	0.00
		LPG	0.92	1.11	0.97	1.22	1.06	1.42
		Electricity	0.00	0.00	0.00	0.00	0.00	0.00
	Lighting	Kerosene	0.10	0.09	0.03	0.02	0.00	0.00
		Electricity and solar	0.00	0.00	0.00	0.00	0.00	0.00
	Space heating	Wood	1.36	1.35	1.29	1.27	0.24	0.17
		Electric & solar	0.00	0.00	0.00	0.00	0.00	0.00
	diesel genset	Diesel	0.21	0.21	0.20	0.20	0.17	0.14
		Electric & solar	0.00	0.00	0.00	0.00	0.00	0.00
Agriculture	Agr.res.burn	Open Residue Burning	2.55	3.12	2.55	3.12	1.58	0.00
		Deep sowing mulching tech	0.00	0.00	0.00	0.00	0.00	0.00
	Agr. Pumps	Diesel	0.19	0.18	0.07	0.04	0.03	0.00
		Electric & solar	0.00	0.00	0.00	0.00	0.00	0.00
	Agr. Tractors	Diesel	0.25	0.30	0.25	0.30	0.22	0.23
		**Total**	**56.64**	**110.84**	**50.23**	**84.75**	**41.14**	**65.00**

S2.5. Technology linked emission factors

For thermal power, emission factors (Table S8) assumed a mean 38% ash content coal, typical of India, with electrostatic precipitators (ESP) working at 99.98% while more efficient supercritical, ultra-super critical and IGCC technologies, had emission reductions in proportion with increased energy efficiency. In December 2015, the Indian Ministry of Environment and Forests issued new norms for thermal plants with emission standards for SO2 and NOx (MoEFCC,2015). Reported barriers to quick adoption of desulphurization and de-NOx technologies (CSE, 2016), lead to assumptions here of low rates of flue gas desulphurization technology adoption. Preliminary surveys show little progress in the implementation of new standards, mainly due to insufficient knowledge in advanced pollution control technologies and lack of i) space for installation, ii) storage for raw materials and iii) clarity on cost recovery (CSE, 2016). Similarly, in heavy industries like cement, iron and steel, fertilizer and non-ferrous, 90% (S1 and S2) and 100% (S3) operation of existing controls are considered while emission factors for PAT technologies were reduced below non-PAT values using their increase in efficiency (Table S8).

It was assumed that non-fired brick production, which uses cement, involves no use of fuel for firing or drying purposes, hence produces no emissions at the stage of brick production, to avoid double-counting of emissions related to feedstock, which are accounted in cement production. In informal industry, the use of traditional biomass technologies for major thermal and drying operations was assumed shift to cleaner gasifier or LPG technologies, hence, emission factors similar to those for residential cooking were considered. In the residential sector, available measurements (reviewed in Pandey et al. 2014) were used to derive emission factors for wood, dung-cake, crop residue combustion in cook stoves, as also for kerosene and LPG cook stoves, which are also used for biomass fired water-heating and space-heating. Diesel generator sets, for residential use and for mobile towers have been included, whose emission factors are set similar to measured factors for agricultural diesel pumps.

In the agriculture sector, emissions from field burning of cereal straw and sugarcane residue were included. Here, emission factors (Table S8) for cereal and sugarcane burning were used, with zero emissions allocated, in cases of future shifts to deep sowing-mulching technology (Gupta, 2014). The distributed diesel category included diesel use in agricultural tractors and pumps, and in diesel generator sets used for non-grid electricity supply. Emission factors for distributed diesel sources are used, with zero emission allocation for a shift to electric or solar technologies.

Table S8. Emission factors of SLCP's and fine particulate matter (g/kg of fuel used)

Sector	Source Categories	TechMix	SO_2	NO_x	NMVOC	$PM_{2.5}$	BC	OC
Thermal power	TPP - coal	Sub-critical	7.3	4.5	0.0	1.8	0.0	0.0
		Super-critical	6.5	4.0	0.0	1.6	0.0	0.0
		Ultra super critical	5.7	3.5	0.0	1.4	0.0	0.0
		IGCC	4.9	3.0	0.0	1.2	0.0	0.0
	TPP - oil & gas	Sub-critical	0.0	3.8	0.0	0.0	0.0	0.0
		Super-critical	0.0	3.4	0.0	0.0	0.0	0.0
		Ultra super critical	0.0	2.9	0.0	0.0	0.0	0.0
		IGCC	0.0	2.3	0.0	0.0	0.0	0.0
Heavy Industry	Cement	PAT	1.2	2.1	0.1	2.3	0.0	0.1
		Non-PAT	1.2	2.1	0.1	2.4	0.0	0.1
	Iron and steel	PAT	5.2	1.9	0.4	1.2	0.3	0.2
		Non-PAT	8.6	3.0	0.7	1.9	0.4	0.3
	Fertilizer	PAT	2.7	1.1	3.7	0.3	0.1	0.0
		Non-PAT	2.7	1.1	3.8	0.3	0.1	0.0
	Non-ferrous	PAT	2.7	1.1	3.7	0.3	0.1	0.0

		Non-PAT	2.7	1.1	3.8	0.3	0.1	0.0
Light Industry		PAT	15.6	2.9	0.0	0.0	0.0	0.0
		Non-PAT	13.5	7.0	0.6	2.4	0.5	0.1
Brick and Informal industry	Brick Production	BTK	9.8	3.8	0.2	3.6	3.1	0.1
		Clamps	9.8	3.8	0.2	4.2	1.6	0.6
		Zig-zag firing	9.8	3.8	0.2	2.0	0.3	0.2
		VSBK	9.8	3.8	0.2	2.3	0.0	1.0
		Non-fired bricks	0.0	0.0	0.0	0.0	0.0	0.0
	Informal industry	Trad. Biofuel	0.4	0.7	11.2	5.6	0.7	2.2
		Gasifier	0.3	0.7	4.3	0.8	0.1	0.3
Transport	Passenger - Private	Gasoline BS III	0.2	32.4	98.4	4.4	0.2	3.5
		Gasoline BS IV	0.2	22.7	68.9	0.9	0.0	0.7
		Gasoline BS V	0.2	13.0	68.9	0.9	0.0	0.7
		Gasoline BS VI	0.2	2.6	19.7	0.4	0.0	0.3
		CNG	0.0	10.8	1.8	0.0	0.0	0.0
		Electric	0.0	0.0	0.0	0.0	0.0	0.0
	Passenger - Public	DieselBS III	0.5	39.2	6.0	6.8	1.2	0.4
		DieselBS IV	0.4	27.5	4.2	1.4	0.2	0.1
		DieselBS V	0.4	15.7	4.2	1.4	0.2	0.1
		DieselBS VI	0.4	3.1	1.2	0.7	0.1	0.0
		CNG	0.0	17.4	0.0	0.0	0.0	0.0
		Electric	0.0	0.0	0.0	0.0	0.0	0.0
	Freight	DieselBS III	0.7	44.9	10.1	6.8	4.4	1.4
		DieselBS IV	0.6	31.5	7.1	1.4	0.9	0.3
		DieselBS V	0.6	18.0	7.1	1.4	0.9	0.3
		DieselBS VI	0.6	3.6	2.0	0.7	0.4	0.1
Residential	Cooking	Trad. Biofuel	0.4	0.7	11.2	5.6	0.7	2.2
		Gasifier	0.3	0.7	4.3	0.8	0.1	0.3
		Kerosene	0.0	0.0	17.0	0.6	0.2	0.3
		LPG	0.3	0.0	18.8	0.3	0.0	0.1
		Electricity	0.0	0.0	0.0	0.0	0.0	0.0
	Lighting	Kerosene	0.0	0.0	0.0	93.0	90.0	0.4
		Electricity and solar	0.0	0.0	0.0	0.0	0.0	0.0
	Space heating	Wood	0.1	0.0	6.9	4.1	0.7	1.9
		Electric & solar	0.0	0.0	0.0	0.0	0.0	0.0
	Diesel genset	Diesel	0.7	97.2	7.7	6.9	4.6	1.5
		Electric & solar	0.0	0.0	0.0	0.0	0.0	0.0
Agriculture	Agr.res.burn	Open Residue Burning	0.5	2.9	13.4	6.1	0.6	2.2
		Deep sowing mulching tech	0.0	0.0	0.0	0.0	0.0	0.0
	Agr. Pumps	Diesel	0.7	97.2	7.7	6.9	4.6	1.5
		Electric & solar	0.0	0.0	0.0	0.0	0.0	0.0
	Agr. Tractors	Diesel	0.7	126.0	1.1	17.0	11.0	4.0

S2.6. Comparison of emissions with other inventories

Comparison with HTAP_v2, REAS2.1 and ECLIPSE

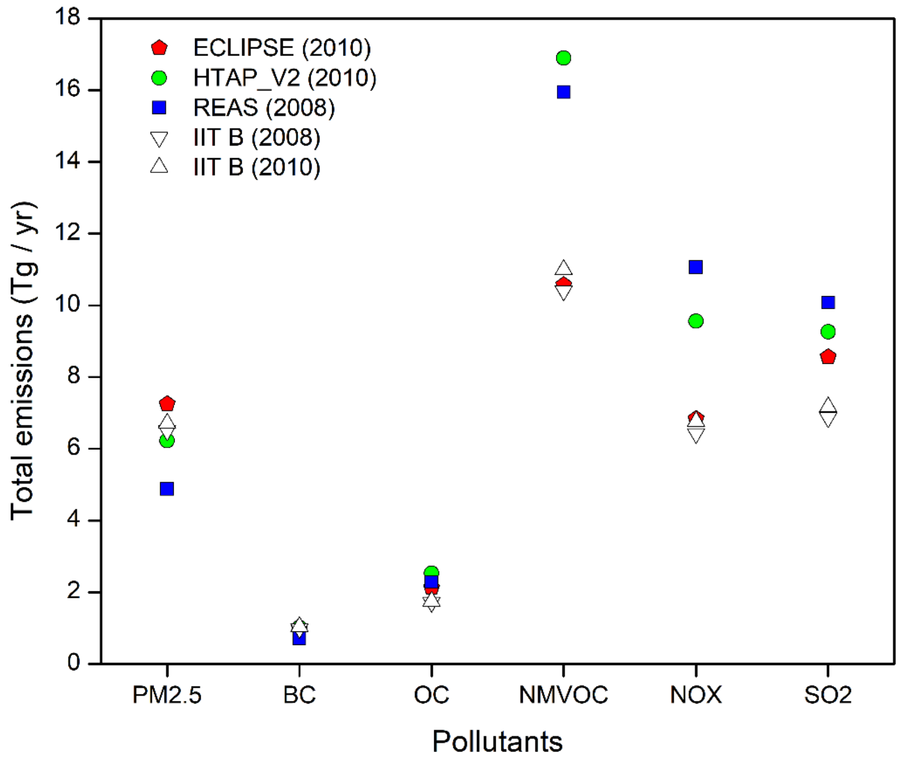

Figure S4. Comparisons of national totals of SLCPs with HTAP_v2, REAS2.1 and ECLIPSE for 2008 and 2010.

The past emissions for 2008 and 2010 are compared to other datasets ECLIPSE (Stohl et al., 2015), HTAP_v2 (Janssens-Maenhout et al., 2015) and REAS 2.1 (Kurokawa et al., 2013). Overall emissions from ECLIPSE were found to be in good agreement with those from our inventory, with the difference in total emissions lying within 20%. However, major differences are found in power generation sector, industry and residential. The differences can be attributed to emissions from extraction processes of fuels, commercial activities, and quantification of process emissions from industries. HTAP agree well with PM and its constituents but is nearly a factor 1.5-2 greater for NOx, NMVOCs and SO2. The differences can be majorly attributed to emissions from extraction process in the power sector and difference in control for NOx and SO2. Similar to HTAP_v2, REAS 2.1 also agrees well for BC and OC while has 0.7 times lower PM and nearly 1.5 times higher emissions of NOx, NMVOCs and SO2 as compared to our inventory. The differences mostly come from inclusion of agricultural emissions (such as fertilizer application and manure management of livestock), non-combustion emissions primarily from solvent use, paint use, evaporative emissions from vehicles, emissions from fuel extraction processes and emissions released from soil in REAS 2.1. Other causes of difference include use of different emission factors and methodologies for emissions estimates, particularly for the residential biomass combustion and transportation. In other inventories, activity data are primarily taken from energy consumption estimates by International Energy Agency (IEA), where as in our inventory the activity data is calculated using food consumption at the state level and end-use energy for cooking (Habib et al., 2004) and vehicular sales to arrive at on-road vehicular population considering age of the vehicles (Pandey and Venkataraman, 2014).

Evaluation with ECLIPSE V5a-CLE and GAINS-WEO2016-NPS

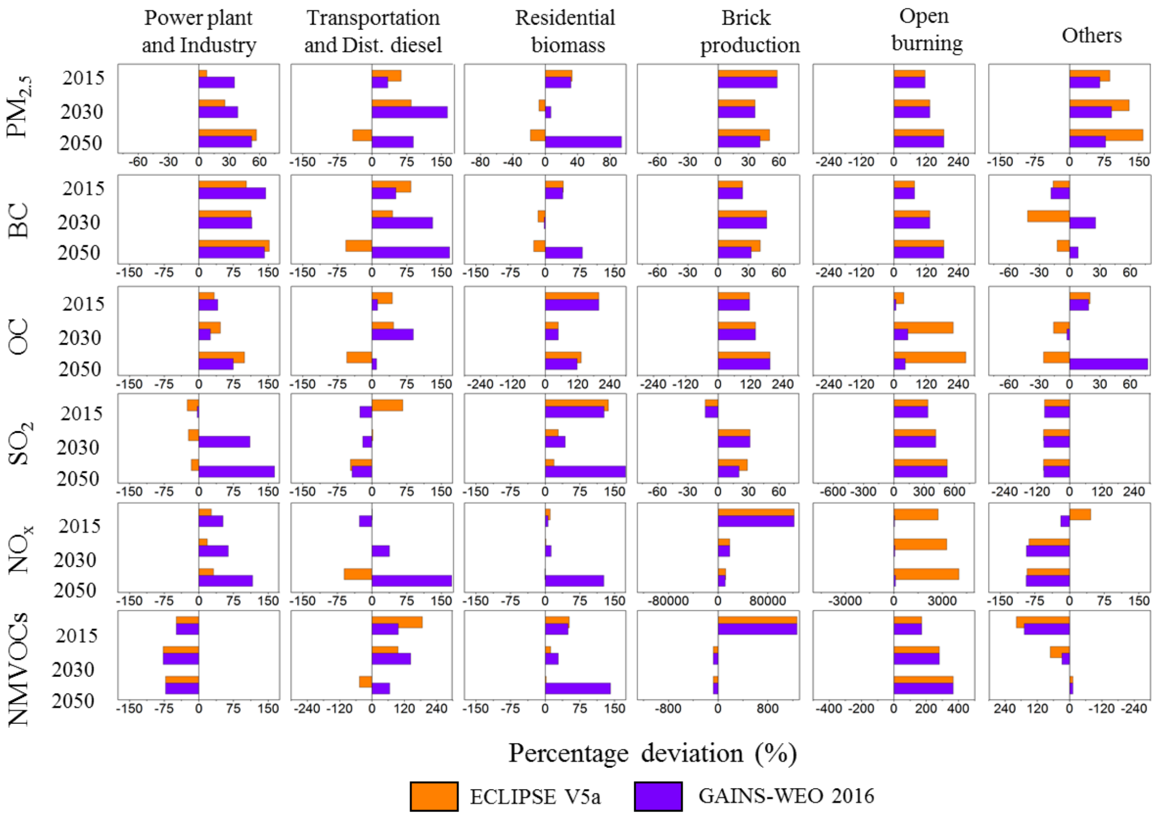

Figure S5. Percentage deviation in emissions of ECLIPSE V5a-CLE and GAINS-WEO2016-NPS from emissions of this study by sector.

(Percentage deviation is calculated as (IITB S2 – ECLIPSE V5a CLE) / ECLIPSE V5a CLE and (IITB S2 – GAINS WEO2016 NPS) / GAINS WEO2016 NPS).

Evaluation with RCP scenarios

RCP2.6 assumes net negative CO2 emissions after around 2070. RCP4.5 and RCP6.0 aim for a smooth stabilization of concentrations by 2150 and RCP8.5 stabilizes concentrations only by 2250. However, RCP scenarios are not tied to any specific socio-economic and technology evolution pathway, making difficult any direct comparison of underlying assumptions, while permitting a comparison of gross emission magnitudes.

Estimated Indian emissions from the RCP scenarios, of SO2, NOx, and NMVOCs, and for BC and OC, for 2005-2050 at 50×50 km resolution, were used for the evaluation. (http://tntcat.iiasa.ac.at/RcpDb/dsd?Action=htmlpage&page=download). The sectors used corresponding to ones in this study, included Energy (Power Plants, Energy Conversion, Extraction, and Distribution), Domestic (Residential and Commercial), Industry (combustion & processing), Surface Transportation and Agriculture waste burning in fields. Gridded emissions in kg m-2 s-1 are summed over the Indian landmass and converted to million tonnes y-1 (Table S9). The present estimates do not include emissions from soils and animal rearing or from shipping and aviation, rather they focus on energy use and solvent based activities. Therefore, corresponding sectors in the RCP database were excluded from the evaluation.

Across RCP scenarios, SO_2 emissions from India are well bounded: 4–9.5 MT/yr in 2030 and 3–7.5 MT/yr in 2050. Emissions of SO_2 estimated here for the highest-control scenario, S3, agreed with those from RCP 4.5 in 2030 and RCP 8.5 in 2050, due to similar assumptions of over 80% non-coal electricity generation. However, the S2 and REF scenarios estimated much larger emissions, respectively, exceeding RCP8.5 by 1.5 to 2 times in 2030 and 3 to 5 times in 2050. This results from our assumption of low levels (max 25%) of deployment of flue gas desulphurization, as only four coal-fired TPPs in India operate flue gas desulphurization (FGD) units and among those to be commissioned through 2030, only 7 TPPs are listed to have FGD (CAT and Urban Emissions, 2014, Prayas Energy Group, 2011), which differs from assumptions of greater SO_2 emission control in the RCP scenarios. These assumptions would reflect in higher secondary sulphate contribution to $PM_{2.5}$ concentrations from thermal and total coal sectors under the REF and S2 scenarios, in 2030 and 2050.

For NO_x emissions as well, there is similar agreement of the S3 scenario here with RCP4.5 in both 2030 and 2050, but significantly larger emissions estimated in the S2 and REF scenarios. The emissions shares are dominated by thermal power and transport sector and grow with sectoral growth under the first two scenarios. Under the S3 scenario, shifts to tighter emission standards for vehicles and a greater share of CNG in public transport, and to non-fossil power generation, reduce NO_x emissions. A non-negligible ~20% share is from residential, agricultural field burning and brick production sectors, which is reduced in magnitude by the adoption of mitigation based largely on cleaner combustion technologies. Similar to emissions of SO_2, those of NO_x in S1 and S2 grow well beyond magnitudes in the RCP database for future years, while those in S3 agree with RCP emission magnitudes, consistent with differences in assumptions in the thermal power sector.

For NMVOC, there is close agreement of S3 scenario emissions with those of RCP6.0 and of S2 scenario emissions lying between those of RCP4.5 and RCP8.5, both in 2030 and 2050. The REF scenario, which assumes negligible shifts away from residential biomass fuel use and agricultural field burning, calculates somewhat larger NMVOC emissions. Present day NMVOC emissions are dominated by residential energy use, largely from traditional biomass fuel stoves, followed by fugitive emissions from energy extraction (coal mining and oil exploration), and open burning of agricultural residues in fields.

Emissions of BC in the S3 scenario agreed best with RCP6.0 in 2030 and RCP8.5 in 2050, while REF and S2 scenario BC emissions exceeded those of the RCP8.5 by factors of 1.5 to 3, from inclusion of new sources like residential lighting (with kerosene wick lamps) and water and space heating (with biomass fuels). Emissions of OC in the S2 scenario closely matched those in RCP4.5, while those in REF matched RCP8.5, in both 2030 and 2050; however, those in S3 were a factor of 3 lower than the lowest RCP6.0 emissions.

Table S9. Representative Concentration Pathways (RCP) scenarios values over India

Scenario	Years	Emissions in MTy^{-1}					
		$PM_{2.5}$	BC	OC	SO2	NOx	NMVOC
**REF**	**2020**	9.8	1.4	2.4	10.3	11.5	15.0
	**2030**	12.0	1.6	2.6	17.8	18.2	14.8
	**2040**	14.5	1.6	2.7	26.8	23.3	15.5
	**2050**	18.5	1.6	2.9	41.4	31.7	16.3
**S2**	**2020**	9.1	1.2	2.2	9.4	10.5	14.1
	**2030**	9.5	1.1	2.2	12.7	13.7	12.5
	**2040**	10.3	1.1	2.2	16.2	15.7	12.4
	**2050**	11.6	1.0	2.2	20.7	18.4	12.4
**S3**	**2020**	6.0	0.9	1.4	6.4	8.6	9.9
	**2030**	3.8	0.5	1.0	6.0	8.6	5.8
	**2040**	3.0	0.3	0.7	6.6	9.2	4.0
	**2050**	3.0	0.3	0.7	7.5	10.5	3.8
**RCP 2.6**	**2020**		0.6	1.9	8.2	4.4	9.9
	**2030**		0.5	1.9	7.1	4.5	10.3
	**2040**		0.4	1.8	4.8	4.8	10.3
	**2050**		0.4	1.5	4.0	5.4	8.5
**RCP 4.5**	**2020**		0.6	1.7	7.9	4.8	12.4
	**2030**		0.7	1.8	8.8	6.0	14.3
	**2040**		0.7	1.8	8.4	6.5	15.8
	**2050**		0.7	1.6	6.8	6.3	16.8
**RCP 6.0**	**2020**		0.4	1.3	5.4	3.1	6.7
	**2030**		0.4	1.3	4.1	2.7	6.1
	**2040**		0.4	1.3	5.3	3.1	5.9
	**2050**		0.4	1.3	5.4	3.3	5.9
**RCP 8.5**	**2020**		0.6	2.0	8.5	5.6	11.0
	**2030**		0.6	2.1	8.8	6.2	12.5
	**2040**		0.7	2.3	9.2	6.1	14.1
	**2050**		0.7	2.5	7.6	4.9	13.2

S3. PM2.5 pollution over India

Top polluted cities in India

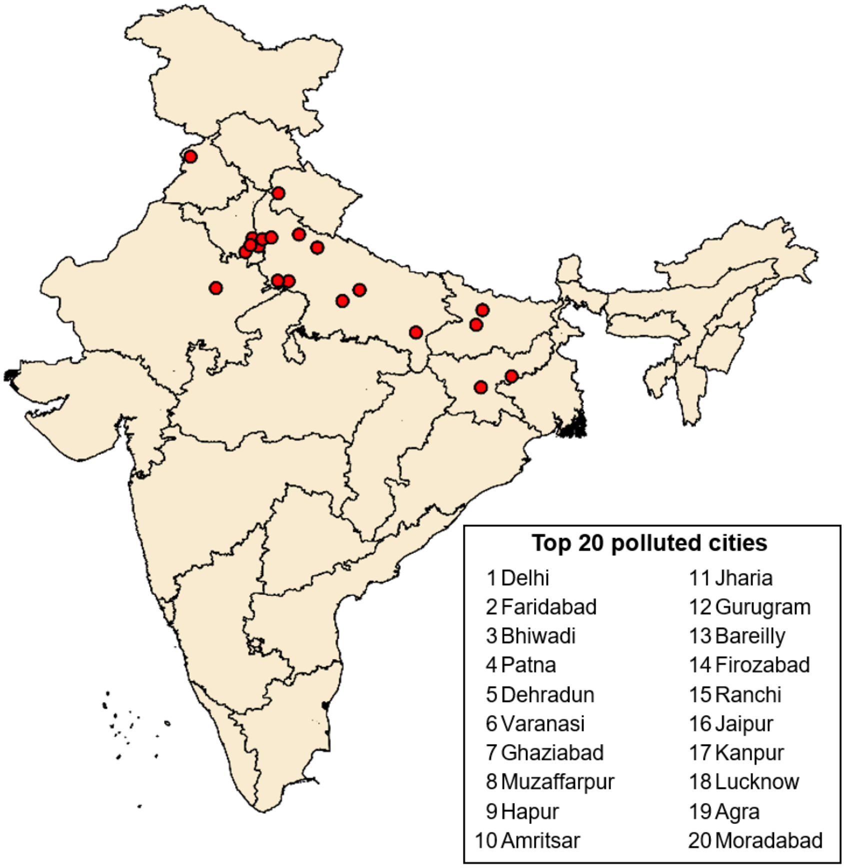

Figure S6. Top 20 polluted cities in India (2016)
(Information taken from Greenpeace, 2018)

NMVOC speciation in GEOS-Chem model

Table S10. Description of GEOS-CHEM NMVOC species

Species in GEOS-Chem	Description
ACET	Acetone
ALD2	Acetaldehyde
ALK4	Lumped ≤ C4 Alkanes
C2H6	Ethane
C3H8	Propane
CH2O	Formaldehyde
MEK	Methyl Ehtyl Ketone
PRPE	Lumped ≤ C3 Alkanes

Mean population weighted ambient PM2.5 concentrations

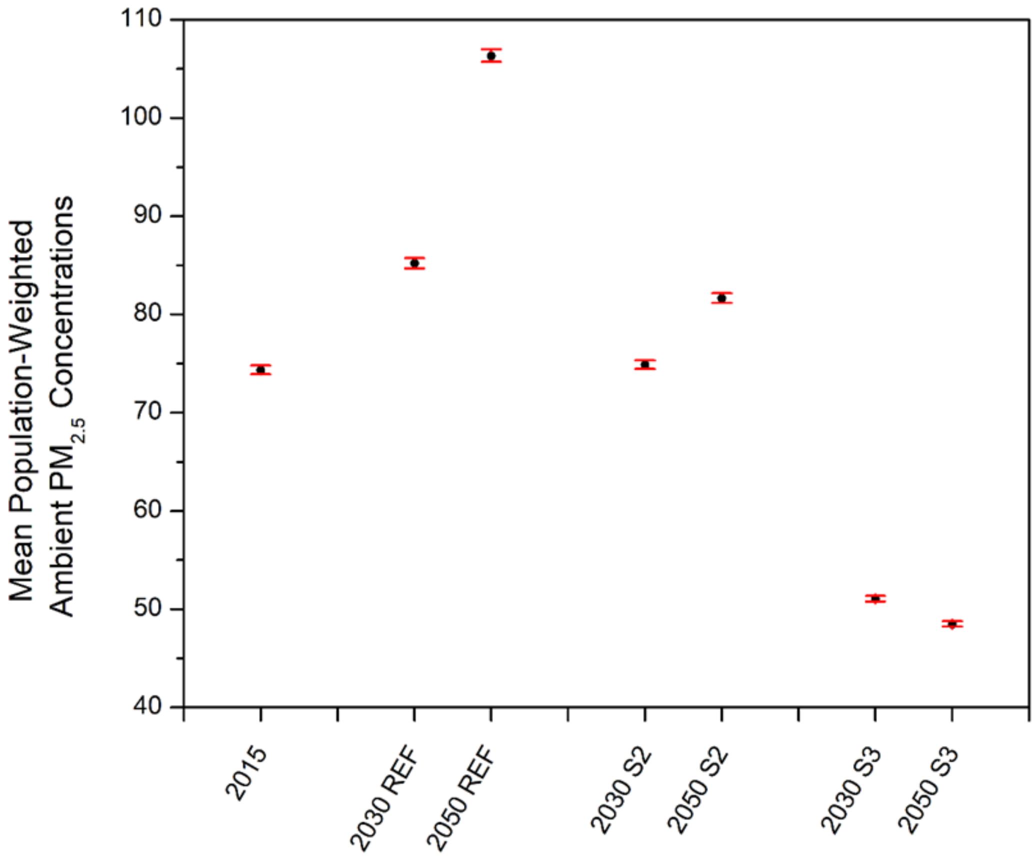

Figure S7. Mean population-weighted ambient PM2.5 concentrations for 2015 and future scenarios. The bars represent the 95% Confidence Interval for the estimates.

The uncertainty represented by the bars is based on uncertainty in the GBD estimates of ambient PM2.5 concentrations. It is estimated by sampling 1,000 draws of a distribution for each grid cell based on the model output mean and standard deviation (GBD MAPS Working Group, 2018).

References

Anandarajah, G. and Gambhir, A.: India's CO2 Emission Pathways to 2050: What Role Can Renewables Play? Applied Energy, 131, 79–86, http://dx.doi.org/10.1016/j.apenergy.2014.06.026, 2014

Auto Fuel Vision and Policy 2025. Report of the Expert Committee: Auto Fuel Vision and Policy 2025.Government of India, http://petroleum.nic.in/docs/reports/autopol.pdf, 2014.

CAT and Urban Emissions: Coal Kills: Health Impacts of Air Pollution from India's Coal Power Expansion, Conservation Action Trust (CAT), Mumbai. http://www.indiaairquality.info/wp-content/uploads/docs/Air%20Pollution%20from%20India%20Coal%20TPPs%20-%20LowRes.pdf, 2014.

CSE: New Environmental Norms for the Power Sector. Proceedings and Recommendations of the Stakeholder Workshop Organized by CSE, Centre for Science and Environment, New Delhi. http://cseindia.org/userfiles/new-environmental-norms-report.pdf, 2016.

DieselNet: Emission Standards, [online] Available from: https://www.dieselnet.com/ (Accessed 17 May 2018), 2018.

EMEP: EMEP/EEA Air Pollutant Emission Inventory Guidebook. European Environment Agency, Copenhagen, 2009.

Firoz, A. S.: Long Term Perspectives for Indian Steel Industry, Ministry of Steel, Economic Research Unit mimeo, New Delhi, May, 2014.

Greenpeace: Airpocalypse II, Assessment of air pollution in Indian cities, 2018.

Gupta, R.: Essays on Air Pollution, Global Warming and Agricultural Productivity. PhD Dissertation, Indian Statistical Institute, New Delhi, 2014.

Guttikunda, S.K., Mohan, D.: Re-fueling road transport for better air quality in India, Energy Policy 68, 556-561, 2014.

ICRA: Indian Automobile Industry: Proposed BS-VI Emission Standards. ICRA Limited, New Delhi, http://www.icra.in/Files/ticker/SH-2016-Q2-6-ICRA-Passenger%20vehicles.pdf, 2016.

IEA (International Energy Agency), (2009). Cement Technology Roadmap. Report by International Energy Agency and World Business Council for Sustainable Development. Pages- 36, https://www.iea.org/publications/freepublications/publication/Cement.pdf. Last accessed 1/April/2016.

IEP: Integrated Energy Policy: Report of the Expert Committee. New Delhi, India. Government of India Planning Commission. Available: http://planningcommission.nic.in/reports/genrep/rep_intengy.pdf, accessed: 14 August 2017, 2006.

IESS, NITI Aayog: India Energy Security Scenarios, 2047, NITI Aayog, Govt of India, Available: http://iess2047.gov.in/, accessed: 1 April 2015, 2015.

IPCC: IPCC Guidelines for National Greenhouse Gas Inventories, Energy, vol. 2, 2006a.

IPCC: IPCC Guidelines for National Greenhouse Gas Inventories, General Guidance and Reporting, vol. 1, 2006b.

Lam, N.L., Chen, Y., Weyant, C., Venkataraman, C., Sadavarte, P., Johnson, M.A., Smith, K.R., Brem, B.T., Arineitwe, J., Ellis, J.E., Bond, T.C., Household light makes global heat: high black carbon emissions from kerosene wick lamps, Environ. Sci. Technol, 46, 13531-13538, 2012.

Li, M., Zhang, Q., Kurokawa, J.-I., Woo, J.-H., He, K., Lu, Z., Ohara, T., Song, Y., Streets, D. G., Carmichael, G. R., Cheng, Y., Hong, C., Huo, H., Jiang, X., Kang, S., Liu, F., Su, H., and Zheng, B.: MIX: a mosaic Asian anthropogenic emission inventory under the international collaboration framework of the MICS-Asia and HTAP, Atmos. Chem. Phys., 17, 935-963, doi:10.5194/acp-17-935-2017, 2017

Maithel, S., Uma, R., Bond, T., Baum, E., Thao, V.T.K., (2012). Brick Kilns Performance Assessment, Emissions Measurements, & A Roadmap for Cleaner Brick Production in India Study report prepared by Green Knowledge Solutions, New Delhi, India. http://www.unep.org/ccac/Portals/50162/docs/Brick_Kilns_Performance_Assessment.pdf. Last accessed: 1/April/2016.

Murthy, K.S.S.: Indian Aluminium Demand to Grow 5 Times by 2030 by Prof. K.S.S. Murthy, Gen. Secretary, Aluminium Association of India, 2014.

MoEFCC: http://pib.nic.in/newsite/PrintRelease.aspx?relid=133726. Press Release. Ministry of Environment, Forest and Climate Change. Government of India, 2015.

MoP: PAT Perform, Achieve and Trade. Ministry of Power, Government of India, 2012.

MoP: Energy Efficient Norms for Power Plants. Press Information Bureau. Ministry of Power. Government of India. Available: http://pib.nic.in/newsite/PrintRelease.aspx?relid=117384, accessed 11 August 2017, 2015.

MoRTH: http://pib.nic.in/newsite/PrintRelease.aspx?relid=134232. Press Release. Ministry of Road Transport and Highways. Government of India, 2016.

National Solar Mission, Ministry of New and Renewable Energy, Govt. of India http://www.mnre.gov.in/solar-mission/jnnsm/introduction-2/, 2010.

NITI Aayog: India Energy Security Scenarios (IESS) 2047. New Delhi: NITI Aayog weblink: http://indiaenergy.gov.in/, 2015

NTDPC: Moving India to 2032, India transport report, Government of India, National Transport Development Policy Committee, India, New Delhi, http://planningcommission.nic.in/reports/genrep/NTDPC_ Vol_01.pdf, accessed: 1 April 2016, 2013.

Pandey, A. and Venkataraman, C.: Estimating emissions from the Indian transport sector with on-road fleet composition and traffic volume, Atmos. Environ., 98, 123–133, doi:10.1016/j.atmosenv.2014.08.039, 2014.

PNGRB: Vision 2030, Natural Gas Infrastructure in India. Report by Industry Group for Petroleum & Natural Gas Regulatory Board, India, 2013.

Prayas Energy Group: Thermal Power Plants on the anvil: Implications and Need for Rationalisation. Pune, India, 2011.

Ray, D.K., Mueller, N.D., West, P.C. and Foley, J.A.: Yield trends are insufficient to double global crop production by 2050. PloS one, 8(6), p.e66428, 2013.

Shukla, P. R., Agarwal, P., Bazaz, A. B., Agarwal, N., Kainuma, M., Masui, T., Fujino, J., Matsuuoka, Y., Hibino, G., Ehara, T.: Low Carbon Society Vision 2050 India, Indian Institute of Management Ahmedabad, National Institute for Environmental Studies, Kyoto University, Mizuho Information & Research Institute, 1–28, http://2050.nies.go.jp/report/file/lcs_asia/indialcs.pdf, 2009.

Shukla, P. R. and Chaturvedi, V.: Low Carbon and Clean Energy Scenarios for India: Analysis of Targets Approach. Energy Economics 34(SUPPL. 3): S487–95. http://dx.doi.org/10.1016/j.eneco.2012.05.002, 2012.

UNDP: Energy Efficiency Improvements in the Indian Brick Industry. Available: www.resourceefficientbricks.org/, United Nations Development Programme, 2009.

Venkataraman, C., Habib, G., Kadamba, D., Shrivastava, M., Leon, J.F., Crouzille, B., Boucher, O. and Streets, D.G.: Emissions from open biomass burning in India: Integrating the inventory approach with high-resolution Moderate Resolution Imaging Spectroradiometer (MODIS) active-fire and land cover data. Global biogeochemical cycles, 20(2), 2006.

Venkataraman, C., Sagar, A.D., Habib, G., Lam, N. and Smith, K.R.: The Indian national initiative for advanced biomass cookstoves: the benefits of clean combustion. Energy for Sustainable Development, 14(2), pp.63-72, 2010.
